# Quercetin as an Agent for Protecting the Bone: A Review of the Current Evidence

**DOI:** 10.3390/ijms21176448

**Published:** 2020-09-03

**Authors:** Sok Kuan Wong, Kok-Yong Chin, Soelaiman Ima-Nirwana

**Affiliations:** Department of Pharmacology, Faculty of Medicine, Universiti Kebangsaan Malaysia, Jalan Yaacob Latif, Bandar Tun Razak, Cheras, Kuala Lumpur 56000, Malaysia; jocylnwsk@gmail.com (S.K.W.); chinkokyong@ppukm.ukm.edu.my (K.-Y.C.)

**Keywords:** bone defect, osteoblast, osteoclast, osteolysis, osteoporosis, quercitrin

## Abstract

Quercetin is a flavonoid abundantly found in fruits and vegetables. It possesses a wide spectrum of biological activities, thus suggesting a role in disease prevention and health promotion. The present review aimed to uncover the bone-sparing effects of quercetin and its mechanism of action. Animal studies have found that the action of quercetin on bone is largely protective, with a small number of studies reporting negative outcomes. Quercetin was shown to inhibit RANKL-mediated osteoclastogenesis, osteoblast apoptosis, oxidative stress and inflammatory response while promoting osteogenesis, angiogenesis, antioxidant expression, adipocyte apoptosis and osteoclast apoptosis. The possible underlying mechanisms involved are regulation of Wnt, NF-κB, Nrf2, SMAD-dependent, and intrinsic and extrinsic apoptotic pathways. On the other hand, quercetin was shown to exert complex and competing actions on the MAPK signalling pathway to orchestrate bone metabolism, resulting in both stimulatory and inhibitory effects on bone in parallel. The overall interaction is believed to result in a positive effect on bone. Considering the important contributions of quercetin in regulating bone homeostasis, it may be considered an economical and promising agent for improving bone health. The documented preclinical findings await further validation from human clinical trials.

## 1. Introduction

The skeletal system is metabolically active and capable of continuous self-renewal in response to biomechanical use, microdamage and fracture [1,2]. The homeostasis of the skeletal system is maintained via concerted communication between bone-building osteoblasts, bone-degrading osteoclasts and mechanosensory osteocytes through a process called bone remodelling. Perturbation of the equilibrium between bone resorption and formation (in the condition of excessive removal of mineralised bone by osteoclasts without the corresponding amount of newly-formed bone by osteoblasts) results in bone loss and delayed bone regeneration [3]. The long-term progression of bone-related disorders generates significant personal impacts and a growing socioeconomic burden due to huge medical costs, sustained disability and reduced quality of life.

The conventional treatment options for osteopenia and osteoporosis consist of antiresorptive agents (such as bisphosphonates, hormone-replacement therapy, selective oestrogen-receptor modulators and anti-RANKL antibodies) and/or anabolic agents (such as intermittent low doses of teriparatide and antisclerostin antibodies) [4,5]. However, these treatment strategies are not free from adverse effects that limit their use. For instance, the use of bisphosphonates and anti-RANKL antibodies is closely associated with gastrointestinal tract symptoms [6]. Hormone-replacement therapy and selective oestrogen-receptor modulators can cause increased risk for venous thromboembolism and cardiovascular events [6,7]. Treatments administered as injections may cause injection-site reactions [5,8]. A prolonged treatment period and high administration dose of teriparatide has been reported to increase the incidence of bone neoplasms [9]. The concerns linked to pharmacological sclerostin inhibition (e.g., romosozumab) include the probability of bone overgrowth and skeletal deformities [5]. Thus, natural compounds with potent bone-conserving properties and fewer side effects could present alternative strategies to overcome the shortcomings of existing therapies.

A wide array of ongoing scientific studies are attempting to develop bone-protecting agents using natural products [10,11,12,13,14]. Quercetin is a natural dietary bioflavonoid present ubiquitously in fruits and vegetables. The reported beneficial effects of quercetin include cardioprotective [15], neuroprotective [16], gastroprotective [17], anticarcinogenic [18], antimicrobial [19], anti-atherosclerotic [20], anti-inflammatory [21,22], antioxidative [21,23], immunomodulatory [22], antihypertensive [24], anti-obesity [25], antihyperglycaemic [26], lipid-modulating [27] and bone-conserving properties [28]. Amongst these, several bioactivities of quercetin make it a potential agent for maintaining bone health.

In this review, the authors provide a comprehensive summary of literature related to the effects of quercetin on bone in animal models. In particular, the underlying cellular and molecular mechanisms of quercetin that can modulate skeletal homeostasis and protect the bones are discussed. 

## 2. In Vivo Evidence on the Effects of Quercetin on Bone

The bone-conserving effects of quercetin have been extensively established using animal models of osteopenia induced by sex-hormone deficiency, streptozotocin (STZ), glucocorticoids, bile-duct ligation, retinoic acid and zinc oxide nanoparticles. Animals with osteolysis, bone defects and healthy animals were also used to examine the protective effects of quercetin on bone (Table 1).

Female rats subjected to bilateral ovariectomy are the most commonly used osteoporotic animal model [54]. Yuan et al. (2018) reported that eight weeks of oral quercetin (50 mg/kg/day) administration to ovariectomised rats increased the bone mineral density (BMD), trabecular number (Tb.N) and trabecular thickness (Tb.Th) of rat femoral metaphysis. Three-point flexural testing indicated increases in elastic and maximum radial degrees as well as elastic and maximum loads of rat femur [29]. Another group of researchers demonstrated that oral supplementation of quercetin (50 mg/kg/day) for 30 days reduced acid phosphatase (ACP) and alkaline phosphatase (ALP) activities, but increased serum calcium and phosphorus levels [30]. In another study, quercetin (2.5%) was added into the diets of female C57BL/6J mice for four weeks after undergoing bilateral ovariectomy. The findings obtained from this study were increases in total lumbar BMD, total femur BMD, cortical bone area (Cr.Ar), cortical thickness (Cr.Th), section modulus, bone volume/total volume (BV/TV), Tb.N, Tb.Th, osteoid volume, osteoid surface and decrease in trabecular separation (Tb.Sp) [31], which indicated that quercetin improved bone density.

Siddiqui and colleagues assessed the bone-sparing effects of quercetin and its structurally related compound, quercetin-6-*C*-β-d-glucopyranoside, in ovariectomised animals. In the first study, adult female rats were given oral 5 mg/kg/day quercetin or quercetin-6-*C*-β-d-glucopyranoside for 12 weeks after bilateral ovariectomy [32]. Quercetin-6-*C*-β-d-glucopyranoside raised BMD level at femur epiphysis, proximal tibia, lumbar vertebra 4 (L4), femur diaphysis and tibia–fibula separating point (TFSP). All trabecular microarchitectural parameters of femur epiphysis and proximal tibia were improved after treatment with quercetin-6-*C*-β-d-glucopyranoside, characterised by higher BV/TV, Tb.Th and Tb.N and lower Tb.Sp. Cortical parameters of femur and tibia were also improved, as shown by increased Cr.Ar, Cr.Th, periosteal area (T.Ar), periosteal perimeter (T.Pm), bone perimeter (B.Pm) and endosteal perimeter (E.Pm). Bone biomechanical strength was increased while bone turnover markers (osteocalcin and C-terminal telopeptide of type 1 collagen (CTX)) were reduced in ovariectomised rats supplemented with quercetin-6-*C*-β-d-glucopyranoside. In contrast, treatment with quercetin resulted in partial maintenance in all these measured parameters [32]. Quercetin and its derivatives differ in the attachment of sugar moieties that affect the pharmacokinetic profile [55]. Hence, a possible explanation for the greater bone-protecting efficacy of quercetin-6-*C*-β-d-glucopyranoside may be that it has better bioavailability and bioactivity as compared to free quercetin. In another study, the same group of researchers administered 5 or 10 mg/kg/day quercetin-6-*C*-β-d-glucopyranoside to ovariectomised rats for 12 weeks [33]. An improvement of bone microstructure at the proximal tibia, diaphysis and epiphysis of the femur was detected. The observation was evidenced by increased BV/TV, Tb.N, Tb.Th, connectivity density (Conn.D), mineral apposition rate (MAR) and bone formation rate (BFR), but decreased Tb.Sp, trabecular pattern factor (Tb.Pf) and structure model index (SMI) [33].

Bone-sparing effects were also observed in the ovariectomised animal model when quercetin was incorporated into nanoparticles or hydroxyapatite bioceramic microspheres. The application of nanoparticles and microspheres has received great attention for their efficient enhancement of bioavailability and bioactivity by prolonging half-life, managing drug particle size and controlling drug release [56,57]. Female albino rats underwent bilateral ovariectomy and were orally treated with 10 or 50 mg/kg quercetin-loaded phytosome nanoparticles for 30 days. The treatment significantly decreased bone turnover markers and increased mineral levels in the sera of the animals [30]. In another study, hydroxyapatite bioceramic microspheres loaded with quercetin were fabricated. It was implanted into a bone defect created at the distal region of the femur diaphysis in ovariectomised rats. It was noted that the BMD and Tb.Th of the bone defects were improved after eight weeks of implantation [34].

On the other hand, the addition of phytochemical blends into animals’ basal diet failed to exert anti-osteoporotic effects in ovariectomy-induced bone loss in aged retired-breeder Fischer 344 rats. Ambati and coresearchers used two types of phytochemical blends containing (a) 1000 mg/kg diet quercetin, 2400 IU/kg diet vitamin D_3_, 500 mg/kg diet genistein and 200 mg/kg diet resveratrol, or (b) 2000 mg/kg diet quercetin, 2400 IU/kg diet vitamin D_3_, 1000 mg/kg diet genistein and 400 mg/kg diet resveratrol. No change was observed in the BMD values or bone microstructure in the femurs of the treated rats after four weeks of feeding with diets containing either intervention [35]. The discrepancy in the outcomes obtained in this study compared to other studies might be because the bone-protecting action of quercetin was inhibited by other compounds.

Quercitrin, a glycoside formed from the flavonoid quercetin and the deoxy sugar rhamnose, has also been investigated for its bone-protecting actions by researchers. Xing et al. (2017) performed bilateral removal of ovaries in female Sprague–Dawley rats and assigned the animals to oral quercitrin (50, 100 or 200 mg/kg/day) treatments for 60 days. The results showed that quercitrin elevated BMD at the distal femur, and increased maximum energy absorption as well as maximum fracture load and stiffness at the femoral neck. Serum analyses of minerals, osteogenic markers and bone turnover markers revealed higher calcium, phosphorus, osterix, runt-related transcription factor 2 (Runx-2), bone formation markers (ALP and N-terminal propeptide of type 1 procollagen (P1NP)) and lower bone resorption markers (CTX and tartrate-resistant acid phosphatase (TRAP)) in the quercitrin-treated ovariectomised rats [36]. Fayed et al. (2019) further delineated the anti-osteoporotic activities of isoquercitrin in ovariectomised rats. The highest dose of isoquercitrin applied in this study (60 mg/kg/day) significantly increased lumbar compression strength. Bone turnover markers were reduced in the isoquercitrin-treated ovariectomised rats [37]. Isoquercitrin was used as previous studies indicated better bioavailability and higher antioxidative activity than quercetin and quercitrin [58,59].

Diabetes mellitus is closely associated with osteopenia, an increased risk of bone fractures and a delay in fracture healing. Therefore, the role of quercetin in diabetic osteopenia was studied by two groups of investigators using an STZ-induced diabetic rat model. In an earlier study, diabetes was induced using a single intraperitoneal (i.p.) injection of STZ at the dose of 50 mg/kg. After eight weeks of diabetes induction, the animals were injected (i.p.) with 15 mg/kg/day quercetin for four weeks. It was noted that quercetin treatment increased BV/TV, Tb.N, Tb.Th, maximum load at femoral mid-diaphysis and neck, and calcium and magnesium concentrations in the diabetic animals. Quercetin treatment also decreased blood glucose level and increased plasma insulin in the STZ-induced diabetic rats [38]. In another experiment, male Sprague–Dawley rats were injected with STZ (100 mg/kg, i.p.) for two consecutive days to induce diabetes, followed by quercetin (30 or 50 mg/kg) treatment after four weeks for a duration of eight weeks. The untreated diabetic rats exhibited higher serum glucose levels and lower bone remodelling compared to the normal controls, as reflected by serum bone markers and histomorphometric evaluation, which subsequently translated to deterioration of bone microstructural and mechanical properties. All these degenerative changes were reversed by the treatment of quercetin [39].

The use of glucocorticoids is one of the most common causes of secondary osteoporosis [60]. Two studies created an osteoporotic model by subcutaneously (s.c.) injecting glucocorticoids in rats. In the first study, methylprednisolone sodium succinate (40 mg/kg) was used as an agent to induce bone loss in female Sprague–Dawley rats. Quercetin at the dose of 150 mg/kg improved bone histomorphometric indices (Tb.Th, Cr.Th osteoblast number and moment of inertia were increased), bone strength and osteocalcin level [40]. More recently, researchers noted that the levels of calcium and phosphorus in serum, as well as weight, density and tensile strength of the femur were significantly raised after topical treatment with quercetin-loaded transfersome film (10 mg/kg) on the dorsal surface for a duration of 15 days. Bone turnover markers were reduced. Histomicrographic examination showed fewer disruptive and lytic changes in the femurs of the treatment group as compared to those without treatment [41].

Besides the more common osteoporotic conditions described above, quercetin has also been studied in osteoporosis related to other stressors. Osteoporosis and increased fracture risk are common complications in patients with primary biliary cholangitis/cirrhosis. Using a rat model of osteoporosis induced by complete bile-duct ligation, the animals receiving quercetin (150 μmol/kg/day) for four weeks showed reversal of the deteriorating effects of cirrhosis on bone strength and histomorphometric indices of trabecular and cortical bones [42]. The use of high-dose vitamin A (retinoic acid) has been reported to cause reductions in BMD and structural bone parameters [61]. Administration of quercetin (100 mg/kg/day) orally via a gastric tube for 14 days increased BMD, ash, calcium and phosphorus content in the femur. Femur weight and femur length were also elevated in the quercetin-treated group [43]. Exposure to zinc oxide nanoparticles caused perturbation of bone turnover, reflected by alteration of bone formation and bone resorption markers. The bone ALP level was higher and serum CTX level was lower in zinc-oxide-nanoparticle-intoxicated rats following treatment with intragastric quercetin (200 mg/kg) for three weeks [44]. Osteolysis is defined by pathological destruction or disappearance of bone tissues. Zhang et al. (2017) determined the protective effects of quercetin against titanium-particle-induced calvarial osteolysis utilising male BALB/c mice. Quercetin at 50 or 100 mg/kg per day inhibited titanium-particle-induced osteolysis by increasing bone area and decreasing osteoclast formation in vivo [45]. In another study, female C57BL/6 mice with titanium-particle-induced osteolysis and treated with quercetin (2 or 5 mg/kg/day) for 14 days had higher BV/TV but lower total porosity, erosion area and osteoclast number [46].

A bone defect is a lack of bone continuity usually caused by trauma, tumour or infection. Quercetin provided beneficial effects on bone defects in rats and rabbits. Song and colleagues created a circular calvarial defect with 4 mm diameter in female Sprague–Dawley rats. The defect was implanted with a quercetin/silk fibroin/hydroxyapatite scaffold with bone-marrow-derived mesenchymal stem cells (MSCs). Six weeks postimplantation, microcomputed tomography results revealed higher values of BMD, BV, BV/TV, BS, Tb.N and Tb.Th. Histological observation also demonstrated that the defect site implanted with bone-marrow-derived MSCs seeded on the quercetin/silk fibroin/hydroxyapatite scaffold had thicker bone matrix, increased formation of new collagenous tissue and tissue ingrowth [47]. A subsequent study explored the effects of quercetin/duck’s foot collagen/hydroxyapatite sponge on calvarial bone defects. Similar outcomes were found, with increased BMD, BV and new bone formation 8 weeks after surgery [48]. In another study, parietal bone defects in New Zealand rabbits were grafted with quercetin solution mixed with collagen matrix for 14 days and bone histological assessment was performed to quantify new bone formation. More new bone was present in quercetin-grafted animals [49,50]. Córdoba et al. (2018) drilled holes and placed four quercetin-nanocoated implants in both tibias of model rabbits. The quercetin implants significantly decreased the expression of osteoclast-related genes (cathepsin K (CTSK), vacuolar type proton ATPase (H^+^ ATPase) and matrix metalloproteinase-9 (MMP-9)). The level of receptor activator of nuclear factor-kappa B ligand (RANKL) was also significantly reduced in rabbits implanted with quercetin-nanocoated implants [51].

Heterogenous findings on bone parameters were obtained in healthy rats upon treatment with quercetin, whereby three studies reported positive outcomes while one study showed a negative impact on bone. In growing rats, higher MAR and BFR were observed at the periosteal and endosteal sites of femur mid-diaphysis in the animals treated with quercetin-6-*C*-β-d-glucopyranoside. Dual-energy absorptiometry and histomorphometry analyses reported higher femoral BMD, tibial BMD, Cr.Ar, Cr.Th, T.Ar, T.Pm, B.Pm and E.Pm at mid-diaphysis and TFSP after quercetin-6-*C*-β-d-glucopyranoside supplementation [33]. In senescence-accelerated OXYS rats, dihydroquercetin (5.06 mg/kg/day) was given in combination with glucosamine alendronate (1.26 mg/kg/day) for two months. The BMD values (total, lumbar and humerus), force applied at failure point and strength of femur were increased, whereas the level of CTX was decreased in the treated ageing rat model [52]. A study by Babosova et al. (2016) also yielded results showing an increase in Cr.Th in healthy 5 month old female rabbits treated intramuscularly (i.m.) with quercetin (10 or 100 μg/kg) three times a week [53]. In contrast, Oršolić et al. (2014) found that quercetin (100 mg/kg/day) given orally for 14 days did not change the weight, length, or ash, calcium and phosphorus content in normal female fertile Y59 rats [43].

Taken together, most of these in vivo studies have shown that quercetin enhances bone quality by improving BMD, trabecular and cortical bone microstructure, bone strength and bone histomorphometric parameters in animals with osteopenia, osteolysis and bone defects. In general, quercetin was administered to animals via oral gavage, injection (i.p. or i.m.) or implantation. Two studies reported paradoxical findings, probably due to the following reasons: Firstly, the beneficial effects of quercetin on preventing bone loss may be inhibited by other phytochemicals, which is worth further validation. Secondly, quercetin may not augment bone health significantly in healthy animals. In view of the potential skeletal benefits of quercetin in vivo, we looked further into the effects of quercetin on bone cells and the molecular mechanisms involved in orchestrating bone metabolism.

## 3. In Vitro Evidence of the Effects of Quercetin on Bone Cells

### 3.1. The Effects of Quercetin on Osteoblastogenesis

In in vitro studies, quercetin has been incubated with MSCs, murine or human osteoblast cells to investigate its effects on cell viability, proliferation, mineralisation and expression of osteogenic genes (Table 2). The proliferation and differentiation of osteoblasts are mainly regulated by a number of transcription factors (osterix, Runx-2, core binding factor alpha 1 (Cbfα1) and type 1 collagen (COL1)), growth factors (bone morphogenetic protein-2 (BMP-2), transforming growth factor-beta 1 (TGF-β1) and vascular endothelial growth factor (VEGF)) and bone markers (ALP, bone sialoprotein (BSP), osteopontin and osteocalcin). Hence, these are the parameters that are commonly measured to determine the osteogenic ability of quercetin.

MSCs are adult stem cells capable of differentiating into osteoblasts, chondrocytes, myocytes, adipocytes, neurocytes and hepatocytes [100]. Using rat or mouse bone-marrow-derived MSCs, quercetin in various concentrations promoted proliferation, differentiation and mineralisation into osteoblastic lineage with a concomitant increase in the expression of ALP, osterix, Runx-2, COL1, BMP-2, BSP, osteopontin, osteocalcin, TGF-β1 and Cbfα1 [34,62,63,64,65]. Adipose-derived stem cells are MSCs derived from adipose tissues. Similarly to MSCs, they are capable of differentiating into multiple cell lineages [101]. In mouse or human adipose-tissue-derived stem cells cultured in osteogenic medium, the presence of quercetin promoted the expression of ALP, osterix, Runx-2, COL1, BMP-2, osteopontin and osteocalcin [66,67]. In addition, quercetin increased cell proliferation, ALP activity, BSP, Runx-2, Cbfα1/Runx-2 and calcium content upon incubation with murine pre-osteoblastic MC3T3-E1 cells [50,68], human osteoblast-like MG-63 cells [69], rat osteoblast-like ROS 17/2.8 cells [70], rat femoral diaphyseal and metaphyseal tissues [71] or osteoblasts derived from rat calvaria and bone marrow [33]. Recently, Lee and colleagues fabricated three-dimensional stem-cell spheroids (containing gingiva-derived stem cells and MC3T3-E1 cells) and cultured them in osteogenic medium in the presence of quercetin at the concentration of 1 μg/mL. Quercetin resulted in significant increases in ALP activity and Runx-2 expression [72]. Human osteoblast-like MG-63 cells grown in medium containing ethanolic fraction of *Cissus quadrangularis* enriched with rutin (65.36 ± 0.75 mg/g) and quercetin (1.06 ± 0.12 mg/g) also displayed higher ALP levels [73].

Inflammation and oxidative stress cause harm to bone cells. The osteogenic activities of quercetin have also been elucidated in cells treated with inflammatory cytokines, lipopolysaccharides (LPS), hydrogen peroxide (H_2_O_2_), menadione and cigarette-smoke medium. Researchers have pointed out that quercetin rescued tumour necrosis factor-alpha (TNF-α)-induced osteogenesis impairment in rat bone-marrow-derived MSCs and MC3T3-E1 mouse pre-osteoblasts [29,74]. Quercetin reversed LPS-stimulated inhibition of osteoblast differentiation by increasing the expression of osteoblast-specific genes such as ALP, osterix, Runx-2, COL1, BSP and osteocalcin [75,76]. Quercetin conferred protection against H_2_O_2_−, menadione- and cigarette-smoking-induced oxidative stress in MC3T3-E1 osteoblast cells and primary human osteoblasts by improving cell viability [77,78].

The effects of quercetin derivatives on osteoblast function in vitro have also been investigated. Quercetin 3-glucuronide (5 μM) upregulated BSP and Cbfα1/Runx-2 in rat osteoblast-like ROS 17/2.8 cells [70]. Quercetin *C*-glucoside (10 or 100 μM) stimulated ALP activity, mineralisation, Runx-2, BMP-2, osteocalcin, and COL1 in rat calvarial osteoblasts and bone marrow cells [33]. Quercetin aglycone (20 μM) increased mineralised nodules, ALP, Runx-2, BSP and osteocalcin in osteoblasts isolated from fetal rat calvaria treated with H_2_O_2_ [80]. Exposure of human osteoblast-like SaOS-2 cells with quercetin 3-β-D-glucoside and polyphosphate caused upregulation of Runx-2 and its co-activators (activating transcription factor 6 (ATF6) and Ets oncogene homolog 1 (Ets1)) [81]. Murine osteoblastic MC3T3-E1 cells exposed to quercitrin had higher cell growth, collagen, ALP, BSP, osteocalcin and mineralisation [82,83].

Comparably, the incorporation of quercetin into biodegradable materials, hydroxyapatite, transition metals or graphene oxide produced anabolic effects via the upregulation of osteogenic genes. Biodegradable poly(lactic-co-glycolic acid)-based microspheres loaded with 1 μg/mL quercetin raised ALP, COL1 and Runx-2 expression in stem-cell spheroids cultured in osteogenic medium [72]. Song and co-authors found that two types of three-dimensional bone scaffold (quercetin/silk fibroin/hydroxyapatite scaffold and quercetin/duck’s foot collagen/hydroxyapatite sponge) enhanced osteogenesis, determined by increases in MSCs’ growth and proliferation, ALP, COL1, osteocalcin and Runx-2 [47,48]. Tripathi et al. (2015) seeded murine MC3T3-E1 cells onto calcium-deficient hydroxyapatite scaffolds with quercetin and found elevations of osteoblast number, ALP, Runx-2, COL1, BSP, osteocalcin and calcium mineralisation [68]. Forte and co-researchers conducted two in vitro studies using an osteoblast–osteoclast co-culture model and osteoblast–osteoclast–endothelial cell triculture model. The co-culture or triculture was seeded on hydroxyapatite loaded with quercetin, and the results indicated higher cell viability and cell proliferation of osteoblasts, expression of ALP, COL1, osteocalcin, Runx-2 and osteonectin [84,85]. The complexes formed by the interaction between quercetin and vanadium or copper were shown to exert osteogenic action, as they stimulated matrix mineralisation, calcium deposition, ALP, COL1, Runx-2 and osteoblast-specific micro-RNA (pre-mir-15b) in murine osteoblastic MC3T3-E1 cells and human osteoblast-like MG63 cells, respectively [86,87]. Chu et al. (2018) constructed polyethylene glycolylated graphene-oxide-mediated quercetin-modified collagen hybrid (ADM-GO-PEG/quercetin) scaffolds with the purpose of efficient therapeutic drug delivery. Results from their study demonstrated that ADM-GO-PEG/quercetin enhanced MSC proliferation and ALP and Runx-2 levels [88]. Seeding of bone-marrow-derived MSCs on a quercitrin-nanocoated titanium surface promoted cell viability, cell adhesion, calcium content and mineralisation [89].

Extensive research has shown favourable properties of quercetin and its related compounds on bone; however, there have been a few studies reporting negative effects. Using rat calvarial osteoblast-like cells exposed to quercetin (1–10 μM), one study reported significant reductions in proliferation, differentiation and mineralisation (as shown by decreased cell number, ALP, osteocalcin, calcium deposition and mineralised nodules) [92]. A study done by Son et al. (2006) reported that quercetin treatment itself did not reduce cell viability and did not exert a substantial cytotoxic effect on MC3T3-E1 cells. The presence of TNF-α decreased cell viability and increased cytotoxicity, but treatment with quercetin caused further acceleration of TNF-α-induced decrease in cell viability and TNF-α-induced cytotoxicity [90]. A recent study by Casado-Díaz et al. (2016) also pinpointed that quercetin inhibited cell proliferation, ALP activity and mineralisation, as well as downregulating markers of osteogenesis (COL1 and osteocalcin) in MSCs induced to differentiate into osteoblasts [93].

In short, the majority of studies have reported osteoblastogenesis-activating effects of quercetin, with a few exceptions. These discrepancies may be related to the cell types and doses of quercetin used. It is important to look further into the mechanisms of action that underlie the osteogenic potential of quercetin.

### 3.2. The Effects of Quercetin on Osteoclastogenesis

Numerous studies have found that quercetin inhibits the formation of osteoclast-like cells (evidenced by TRAP-positive multinucleated cells), bone resorption pit and F-actin ring formation in RAW264.7 cells, human peripheral-blood mononuclear cells (PBMCs) or bone-marrow macrophages treated with macrophage colony-stimulating factor (M-CSF) and/or RANKL [31,32,45,46,68,74,94,95,96]. In another study using highly purified rabbit osteoclasts, treatment with 50 μM quercetin caused a reduction in the total area of resorption pit [97]. The measurement of hydroxylysylpyridinoline, a collagen cross-link molecule released during bone resorption, was used by researchers to confirm the bone resorption activity in cultures [97]. Quercetin was found to decrease hydroxylysylpyridinoline content in human PBMCs treated with M-CSF and RANKL, as well as in highly purified rabbit osteoclasts [96,97]. Quercetin at a dose of 15, 25 or 50 μM also blocked LPS-induced osteoclast formation and viability in RAW264.7 cells [98]. Mouse bone-marrow cells cultured in the presence of parathyroid hormone (PTH), a bone-resorbing factor, caused an increase in osteoclast-like cell formation. This increase was suppressed in the presence of quercetin (0.01–1 μM) [71]. Similarly, other structurally similar derivatives of quercetin, such as quercetin 3-*O*-β-D-glucuronide, quercetin-6-*C*-β-d-glucopyranoside and quercitrin, decreased the number of TRAP-positive cells and bone resorption pit area in RANKL-induced osteoclast differentiation in RAW264.7 cells and bone-marrow macrophages [31,32,83]. Another cell culture study evaluated the effects of calcium-deficient hydroxyapatite loaded with quercetin on cell proliferation in RAW264.7 cells exposed to RANKL. Their findings indicated a dramatic reduction in cell number and osteoclast proliferation [68]. Two recent studies demonstrated a reduction in cell viability and proliferation of osteoclasts when co-culturing osteoblast–osteoclast or triculturing osteoblast–osteoclast–endothelial cells on hydroxyapatite loaded with quercetin [84,85]. In summary, the current literature demonstrated that quercetin consistently has inhibitory effects on osteoclast formation, proliferation and maturation.

## 4. The Underlying Mechanisms of Action of Quercetin as a Bone-Protecting Agent

### 4.1. Regulation of the Receptor Activator of Nuclear Factor-Kappa B (RANK)/RANKL/Osteoprotegerin System

Osteoclastogenesis is a multistep process that requires the commitment of osteoclast progenitors and two haematopoietic factors, M-CSF and RANKL. Osteoclastogenesis is an osteoblast- and osteocyte-mediated process wherein these bone cells are the main source of osteoprotegerin and RANKL governing the RANK/RANKL/osteoprotegerin system. RANKL has a pivotal role in activating RANK expressed on osteoclasts and its precursors, subsequently supporting pre-osteoclast differentiation and osteoclast maturation and survival [102]. Osteoprotegerin, a decoy receptor for RANKL, plays an anti-osteoclastogenic role by blocking the RANK–RANKL interaction. The completion of osteoclast proliferation, differentiation and maturation mediated by RANK–RANKL recognition requires sequential recruitment of tumour necrosis factor receptor-associated factor 6 (TRAF6) and activation of downstream signalling molecules (such as mitogen-activated protein kinase (MAPK), nuclear factor-kappa B (NF-κB) and activator protein (AP-1)), followed by the transcriptional amplification of various genes encoding for cellular proto-oncogene (c-Fos), nuclear factor of activated T-cells cytoplasmic 1 (NFATc1), TRAP, CTSK, calcitonin receptor (CalcR), alpha v integrin, MMP-9 and H^+^ ATPase [103]. Dendritic-cell-specific transmembrane protein (Dc-Stamp) is also a key regulator for osteoclast differentiation, wherein its mechanism of action is involved in mediating cell–cell fusion and formation of fully functional osteoclasts [104].

**Figure 1:**
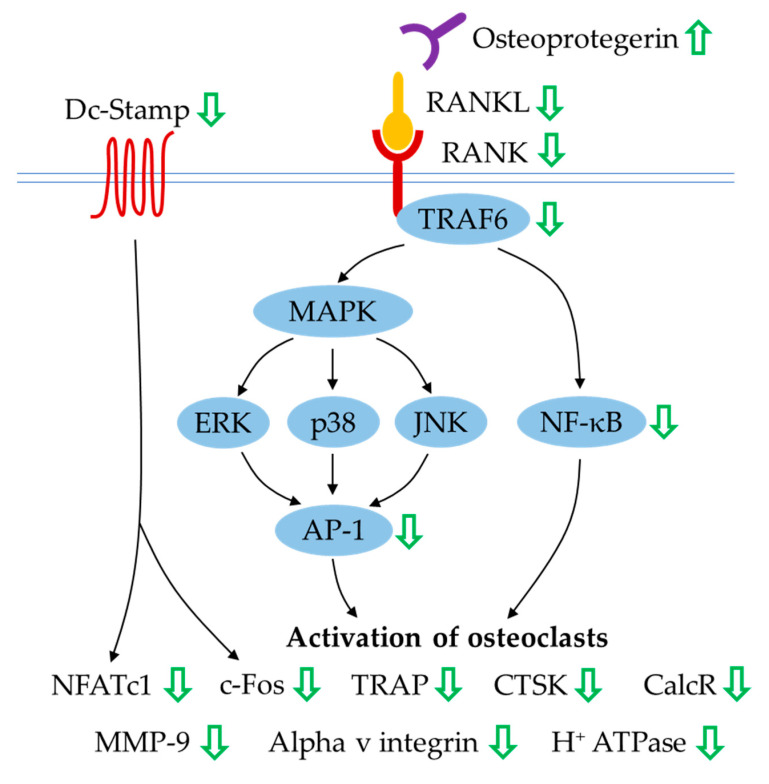
The effects of quercetin on the RANK/RANKL/osteoprotegerin system in regulating osteoclastogenesis. Quercetin causes differential expression of osteoprotegerin and RANKL by osteoblasts. In osteoclasts, quercetin reduces the expression of osteoclast-related markers governed by inhibition of NF-κB and AP-1 activation (indicated by the green arrows); thus, the maintenance of osteoclast lineage commitment, osteoclast maturation and bone resorption are inhibited. Abbreviation: AP-1 = activator protein-1; CalcR = calcitonin receptor; c-Fos = cellular proto-oncogene; CTSK = cathepsin K; Dc-Stamp = dendritic cell-specific transmembrane protein; ERK = extracellular signal-regulated kinase; H^+^ ATPase = vacuolar type proton ATPase; JNK = c-Jun N-terminal kinase; MAPK = mitogen-activated protein kinase; MMP-9 = matrix metalloproteinase-9; NFATc1 = nuclear factor of activated T-cells cytoplasmic 1; NF-κB = nuclear factor-kappa B; RANK = receptor activator of nuclear factor-kappa B; RANKL = receptor activator of nuclear factor-kappa B ligand; TRAF6 = tumour necrosis factor receptor-associated factor 6; TRAP = tartrate-resistant acid phosphatase.

The effects of quercetin on the expression of osteoprotegerin and RANKL have been widely demonstrated. In the osteoblast-like cells, the differential expression of osteoprotegerin and RANKL was observed (where the osteoprotegerin level was increased or unchanged but the RANKL level was decreased), resulting in an increase in osteoprotegerin/RANKL ratio in the presence of quercetin [34,65], quercitrin [82,83] or extract enriched with quercetin [73]. A similar trend of osteoprotegerin and RANKL expression along with the decrease in CTSK was seen when osteoblast–osteoclast–endothelial cell triculture was seeded on hydroxyapatite loaded with quercetin. [85]. In RAW264.7 cells or bone-marrow macrophages treated with M-CSF and RANKL to generate osteoclast-like cells, it was found that expression of osteoclast markers (RANK, c-Fos, NFATc1, TRAP, CTSK, CalcR, alpha v integrin, MMP-9, H^+^ ATPase and Dc-Stamp) was inhibited by quercetin [31,32,45,46,94,95,96], quercetin-6-*C*-β-d-glucopyranoside [32], quercetin-loaded hydroxyapatite [68], quercitrin [83] and quercitrin-nanocoated implant surfaces [51]. Likewise, treatment with quercetin yielded downregulation of TRAP, MMP-9, CTSK, RANK and TRAF6 expression in RAW264.7 cells stimulated by LPS. [98]. The underlying mechanisms of quercetin’s action in orchestrating osteoclastogenesis and bone resorption have been elucidated (Figure 1). The suppression of osteoclastic differentiation and formation induced by flavonoid quercetin is mediated through the inhibition of NF-κB and AP-1 activation [74,96]. In femur bone sections from ovariectomised rats treated with isoquercitrin, immunohistochemical staining of NF-κB in osteoclasts showed lower density than in untreated negative controls [37].

### 4.2. Regulation of MAPK Signalling

The MAPKs are a set of serine/threonine kinases that function as key signal transducers in converting a wide range of extracellular stimuli to cellular responses. In general, activation of the MAPK cascade occurs via the consecutive phosphorylation of three protein kinases. Upon the arrival of a stimulus, mitogen-activated protein kinase kinase kinase (MAP3K) is activated to phosphorylate mitogen-activated protein kinase kinase (MAP2K) and, in turn, phosphorylate MAPK [105]. The three best-studied subgroups of MAPK signalling pathways include extracellular-signal-regulated kinase (ERK), p38 MAPK and c-hJun N-terminal kinase (JNK) signalling pathways, which have been recognised to be critical in modulating the differentiation and activation of both osteoblasts and osteoclasts. ERK1/2 is activated by growth factors, hormones and pro-inflammatory cytokines, whereas p38 MAPK and JNK are activated by pro-inflammatory mediators and cellular and environmental stresses.

Previous studies have confirmed the direct relationship of ERK and p38 MAPK activation with osteogenesis [106,107,108]. Deletion of *Mek1* and *Mek2* (the upstream kinases of ERK signalling) in mice was correlated with lower bone mass and decreased expression of osteoblast-specific genes (Runx-2, ATF4 and β-catenin) [107]. Furthermore, oestrogen-receptor signalling can activate ERK signalling in many cells, including osteoblasts [109], and p38-mediated phosphorylation promotes the expression of osteoblast master regulators like Runx-2, Dlx5, osterix and ATF4 [108]. In contrast, the role of JNK in osteogenesis regulation remains inconsistent; its activation may positively or negatively regulate osteogenesis. The increase of JNK phosphorylation enhanced ALP activity and mineralisation and increased cellular responsiveness to BMP-2 [110,111]. On the other hand, another study hinted at a reverse association between JNK and osteoblastic differentiation. A loss of function in JNK enhanced ALP expression and mineralisation, while the gain of function in JNK reduced BMP-2-mediated osteoblastic differentiation [112]. Activation of JNK and p38 MAPK also induced apoptosis in many cell types, including osteoblasts and osteoclasts [113,114].

Consistently, increased phosphorylation and activation of ERK1/2 was observed in rat bone-marrow-derived MSCs, human adipose-tissue-derived stromal cells, human osteoblast-like MG-63 cells and murine osteoblastic MC3T3-E1 cells incubated with quercetin [34,62,63,67,69,75,76,86]. The data presented by Prouillet et al. (2004) showed that the activation of ERK by quercetin was most likely downstream of the oestrogen-receptor activation [69]. Quercetin exerted dual actions on p38 MAPK to promote osteogenic proliferation and differentiation in MSCs upon its activation, as well as suppressing apoptosis in MC3T3-E1 cells following its inhibition [34,62,63,75]. For the JNK pathway, some studies have reported that quercetin might activate, inhibit or exert negligible effects on osteoblasts. Although the exact mechanism of action of quercetin on JNK signalling in osteoblasts is inconclusive, its net effect has been shown to favour osteogenesis [34,62,63,76]. On the other hand, the activation of JNK pathway by quercetin was associated with acceleration of apoptosis in both osteoblast- and osteoclast-like cells. Quercetin triggered increases in the phosphorylated form of JNK and caspase activation in MC3T3-E1 cells treated with TNF-α, indicating TNF-α-mediated apoptosis via activation of JNK pathway [91]. A study done by Guo et al. (2012) pointed out that quercetin increased protein levels of p-p38 MAPK and p-JNK, thereby increasing Bcl-2-associated X protein (Bax) and decreasing B-cell lymphoma 2 (Bcl-2) in RAW264.7 cells treated with LPS. These findings suggested the augmentation of LPS-induced osteoclast apoptosis upon activation of p38 MAPK and JNK apoptotic signalling pathways [98].

**Figure 2:**
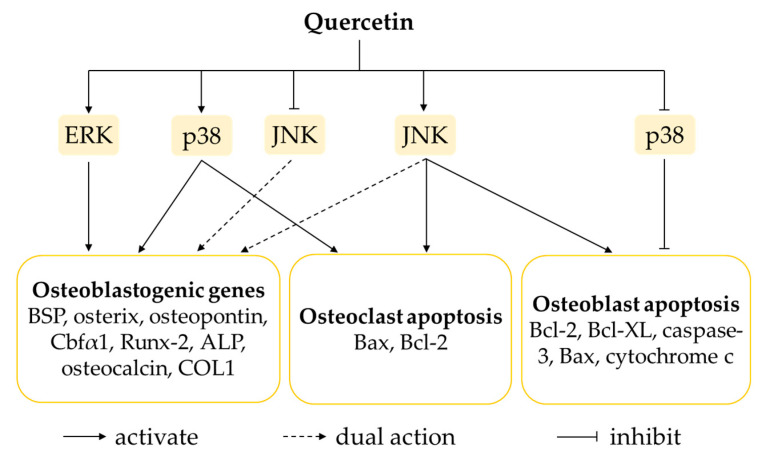
The effects of quercetin on MAPK (ERK, p38 MAPK and JNK) signalling pathways. The activations of ERK and p38 MAPK favour osteogenesis, whereas the activation of JNK positively and negatively regulates osteogenesis. The activations of p38 MAPK and JNK contribute to the induction of osteoblast and osteoclast apoptosis. Quercetin activates ERK but exerts a dual action on p38 MAPK and JNK. Abbreviation: ALP = alkaline phosphatase; Bax = Bcl-2-associated X protein; Bcl-2 = B-cell lymphoma 2; Bcl-XL = B-cell lymphoma-extra large; BSP = bone sialoprotein; Cbα1 = core binding factor alpha 1; COL1 = type 1 collagen; ERK = extracellular signal-regulated kinase; JNK = c-Jun N-terminal kinase; Runx-2 = runt-related transcription factor 2.

The regulation of osteoblastogenesis and osteoclastogenesis governed by MAPK signalling pathways is highly complex (Figure 2). The current state of knowledge shows that even though quercetin has biphasic effects on activation or inhibition of MAPKs (particularly p38 MAPK and JNK), the net outcomes obtained are the enhancement of osteoblast function and osteoclast apoptosis, as well as the suppression of osteoblast apoptosis.

### 4.3. Regulation of Apoptosis

Apoptosis is a process of programmed cell death mediated by two main apoptotic pathways, i.e., the intrinsic (mitochondria-dependent) pathway and extrinsic (death-receptor-mediated) pathway. The mitochondrial pathway is driven by the presence of intracellular cues such as cellular damage and oxidative stress. These stimuli downregulate anti-apoptotic (Bcl-2 and B-cell lymphoma-extra large (Bcl-XL)) and upregulate pro-apoptotic (Bax and Bcl-2 homologous antagonist/killer (Bak)) molecules, leading to the mitochondrial release of cytochrome c. The binding of cytochrome c with apoptotic protease activating factor-1 (Apaf-1), deoxyadenosine triphosphate (dATP) and procaspase-9 forms the apoptosome, catalysing the conversion of procaspase-9 to caspase-9. Eventually, the downstream effector caspases (caspase-3 and caspase-7) are activated, acting as a molecular switch towards cell apoptosis [115]. In the extrinsic pathway, two examples of death ligands are Fas ligand (FasL) and tumour necrosis factor-related apoptosis-inducing ligand (TRAIL), recognised by their receptors, the Fas and TRAIL receptors. Their interaction results in the formation of the death-inducing signalling complex (DISC), consisting of Fas-associated death domain (FADD) and caspase-8, to activate downstream effector caspase-3, which further induces typical apoptosis features including cell fragmentation and cell death [116].

Quercetin has been demonstrated to inhibit cell growth via promoted cell apoptosis in mouse embryo 3T3-L1 pre-adipocytes [117]. Increases in lactate dehydrogenase (LDH) activity (a marker of cell-membrane damage), number of apoptotic cells and collapse of mitochondrial membrane potential have been evidenced. Quercetin also modulates apoptosis-related activity and proteins by downregulating poly(ADP-ribose) polymerase (PARP) and Bcl-2 and upregulating caspase-3 activity, Bax and Bak [117]. On the other hand, quercetin protected murine osteoblastic MC3T3-E1 cells against LPS-induced apoptosis. Pretreatment with quercetin restored the downregulated Bcl-2 and Bcl-XL expression as well as the upregulated caspase-3, Bax and cytochrome c expression caused by LPS [75]. Zhang et al. (2017) reported that quercetin had anti-apoptotic effects in vitro and in vivo, shown by inhibition of titanium-particle-induced endoplasmic-reticulum-stress-related cell apoptosis. The levels of protein kinase RNA-like endoplasmic reticulum kinase (PERK), inositol-requiring enzyme 1 (IRE1), glucose-regulated protein (GRP78), CCAAT/enhancer-binding protein homologous protein (CHOP), caspase-12 and caspase-3 were decreased, whereas Bcl-2 level was increased in the titanium-particle-treated RAW264.7 cells and titanium-particle-implanted calvarium. [45]. Seeding the co-culture of osteoblasts and osteoclasts onto hydroxyapatite loaded with quercetin resulted in the lowering of LDH activity in osteoblasts and upregulation of caspase-3 in osteoclasts [84]. Quercetin also increased the number of apoptotic osteoclasts in highly purified rabbit osteoclast cultures in a dose-dependent manner [97].

**Figure 3:**
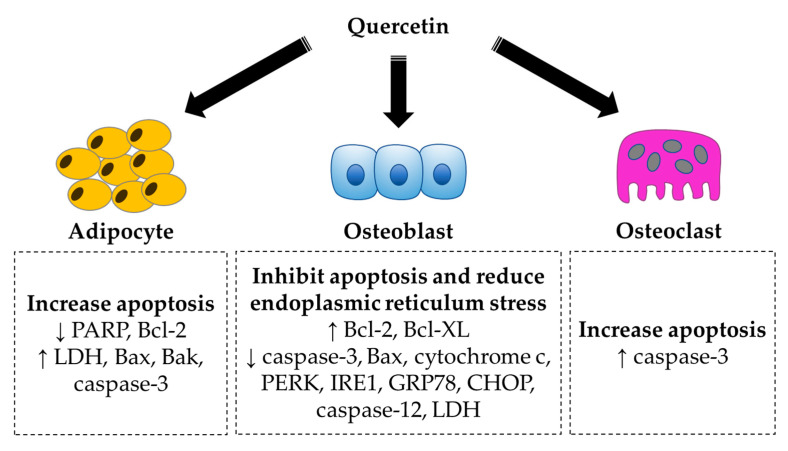
The regulation of apoptosis by quercetin in bone cells. Quercetin induces apoptosis in adipocytes and osteoclasts. In contrast, quercetin inhibits apoptosis and reduces endoplasmic-reticulum-stress-related cell apoptosis in osteoblasts. These actions favour bone formation, thus suggesting the bone-protecting effects of quercetin. Symbol ↑ indicates an increase or upregulation and ↓ indicates a decrease or downregulation. Abbreviations: Bak = Bcl-2 homologous antagonist/killer; Bax = Bcl-2-associated X protein; Bcl-2 = B-cell lymphoma 2; Bcl-XL = B-cell lymphoma-extra large; CHOP = CCAAT/enhancer-binding protein homologous protein; GRP78 = glucose-regulated protein; IRE1 = inositol-requiring enzyme 1; LDH = lactate dehydrogenase; PARP = poly(ADP-ribose) polymerase; PERK = protein kinase RNA-like endoplasmic reticulum kinase.

In short, quercetin plays a role in inhibiting apoptosis in osteoblasts but promoting apoptosis in adipocytes and osteoclasts (Figure 3). Osteoblasts and adipocytes share a common mesenchymal progenitor. The increase in osteoblast formation decreases the MSC reserve for adipocyte differentiation.

### 4.4. Antioxidative Effects

The physiological intracellular redox balance is delicately regulated, with the production of reactive oxygen species (ROS) or/and reactive nitrogen species (RNS) neutralised by an interacting network of antioxidants in the body. Mitochondria are the primary site of production for ROS. The synthesis of superoxide anions (O_2_•^−^) during oxidative phosphorylation is converted to hydrogen peroxide (H_2_O_2_) by superoxide dismutase (SOD). The less-reactive H_2_O_2_ can be further detoxified by glutathione peroxidase (GPx) or/and catalase (CAT) to form water. If antioxidants do not inactivate H_2_O_2_, it can be subjected to photoexcition to generate hydroxyl radicals (OH•) [118,119]. On the other hand, the synthesis of RNS is initiated in the production of nitric oxide (NO•) by nitric oxide synthase (NOS). The reaction between NO• and O_2_•^−^ forms a stronger oxidant, the peroxynitrite anion (ONOO^−^). Subsequently, ONOO^−^ reacts with other molecules to form other RNS such as nitrogen dioxide (NO_2_•) and dinitrogen trioxide (N_2_O_3_) [120]. The supraphysiological levels of ROS or/and RNS and depletion of antioxidant capacity result in oxidative and nitrosative stress that severely damages lipids, proteins and deoxyribonucleic acid (DNA). Due to the short life of ROS, oxidative stress is usually measured by tracing the level of modified oxidative products such as lipid peroxides (malondialdehyde (MDA)), oxidised proteins (nitrotyrosine and protein carbonyl), oxidised nucleic acid bases (8-hydroxy-2′-deoxyguanosine (8-OHdG)) and enzymatic markers (myeloperoxidase) [121].

The major signalling pathway that responds to oxidative and nitrosative stress is the nuclear factor erythroid 2-related factor 2 (Nrf2) pathway. The key signalling molecules, Nrf2 binds to Kelch-like ECH-associated protein 1 (Keap1) in the cytoplasm to form an inactive complex under basal conditions, thus suppressing its transcriptional activity. Under oxidative conditions, Nrf2 dissociates from Keap1, allowing its translocation into the nucleus, followed by heterodimerisation with small musculoaponeurotic fibrosarcoma oncogene homolog (Maf) proteins and association with antioxidant-responsive element (ARE). Upon binding, the expression of downstream Nrf2 target cytoprotective genes and antioxidative enzymes (including heme-oxygenase 1 (HO-1), nicotinamide adenine dinucleotide phosphate quinone dehydrogenase 1 (NQO1), γ-glutamyl-cysteine ligase catalytic subunit (GCLC), glutathione-S-transferase (GST), SOD, CAT and reduced glutathione (GSH)) are driven to circumvent oxidative and nitrosative insult [122,123]. Apart from that, there are molecular interactions between Nrf2 and other signalling proteins like ERK and NF-κB. The activation of the ERK signal transduction pathway regulates Nrf2-dependent transcription by promoting the release of Nrf2 from Keap1 [124]. In contrast, Nrf2 signalling has an opposing effect on NF-κB activity, whereby the activation of Nrf2 pathway inhibits NF-κB by upregulating antioxidant defence and HO-1 expression [125].

In experimental in vitro studies, researchers have found that the antioxidative properties of quercetin and its related glycoside contribute to their osteoblastogenic and anti-osteoclastogenic properties. Primary human osteoblasts exposed to cigarette-smoke medium rapidly produced ROS, leading to a decrease in cell viability. Incubation with quercetin reversed these effects by increasing the expression of antioxidative enzymes such as HO-1 and SOD [78]. Another study showed that treatment of co-culture of osteoblasts and osteoclasts with H_2_O_2_ induced the generation of ROS. The elevation in ROS level was prevented when the co-culture was seeded on hydroxyapatite loaded with quercetin in the presence of H_2_O_2_ [84]. Quercitrin also provided protective effects against H_2_O_2_-induced oxidative stress in osteoblastic MC3T3-E1 cells, whereby the levels of ROS-damaged biological molecules such as MDA, protein carbonyl and nitrotyrosine were reduced [82]. Messer and collaborators reported that osteoblasts derived from fetal rat calvaria treated with quercetin aglycone showed marked upregulation of HO-1, CAT, GCLC and peroxiredoxin-5 (Prdx5) [79]. In the subsequent year, the same group of investigators cultured osteoblasts from fetal rat calvaria in the presence of an oxidative stressor (H_2_O_2_) and treated them with quercetin aglycone. Sustained upregulation of HO-1 and GCLC was observed in osteoblasts without treatment, but it was blocked by the addition of quercetin aglycone [80]. At this juncture, it might be postulated that these increases in HO-1 and GCLC expression might be due to the need to counteract the overwhelming oxidative response induced by H_2_O_2_. The decreases in antioxidant gene expression upon quercetin aglycone incubation indicate the role of quercetin in maintaining a balanced profile between oxidative stress and antioxidant capacity.

The findings of mechanistic studies conducted by researchers remain inconsistent. Quercetin protected the primary human osteoblasts from toxic effects of oxidative stress via the Nrf2 and ERK signalling pathways in one study. Increased phosphorylation of Nrf2 and ERK1/2 in osteoblasts became evident upon stimulation with quercetin [78]. However, a study by Messer et al. (2015) showed no alteration in Nrf2 accumulation in the nucleus and cytoplasm of fetal rat calvarial osteoblasts, even though there was upregulation of antioxidant genes. The authors postulated that the role of the Nrf2 signalling pathway in controlling the transcription of antioxidant genes might be complex, whereby multiple post-translational modifications of Nrf2 and Kaep1 might be involved. In addition, they also reported that the upregulation of antioxidant genes was associated with suppressed phosphorylation of ERK1/2, which was in direct contrast with the findings from a study by Braun et al. (2011). The authors suggested that the effects of quercetin might be different depending on the cell and culture conditions. For NF-κB activity, there was a downregulation in the total level of NF-κB p65 in osteoblasts after treatment with quercetin aglycone [79].

Quercetin also attenuated oxidative and nitrosative stress in osteoclasts. As reported by Wattel et al. (2003), the intracellular ROS production in highly purified rabbit osteoclasts was reduced after treatment with quercetin [97]. Later on, Tang et al. (2019) indicated that both quercetin and quercitrin reduced the LPS-stimulated elevation in NO• and ROS production in RAW264.7 [99]. In the following year, quercetin was shown to inhibit the expression of inducible nitric oxide synthase (iNOS) in RAW264.7 cells treated with M-CSF and RANKL [46].

Several in vivo studies have reported these oxidative-stress-prevention effects alongside the bone-protecting actions of quercetin. Oral administration of free quercetin and quercetin-loaded phytosome nanoparticles reduced MDA level and increased GSH content in ovariectomised rats [30] and in retinoic-acid-induced bone loss model [43]. The concentration of urinary 8-OHdG was lowered, whereas the serum total antioxidant capacity, SOD, GPx, CAT and GST were increased in STZ-induced diabetic rats orally treated with quercetin for 8 weeks [39]. Along with the elevation of bone formation markers and reduction in bone resorption markers, Abdelkarem et al. (2016) also found a decrease in NO• and DNA damage in rats exposed to zinc oxide nanoparticles and treated with quercetin [44]. Nonetheless, no effect was observed in oxidative-stress biomarkers and antioxidant activities when quercetin was orally supplemented to healthy rats [43].

**Figure 4:**
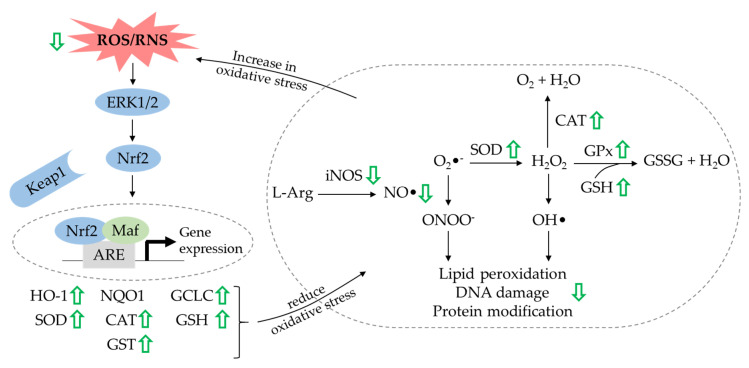
The effects of quercetin on the regulation of oxidative stress in protecting bone. Quercetin decreases oxidative stress and upregulates the expression of antioxidants in osteoblasts and osteoclasts (indicated by the green arrows). Abbreviations: ARE = antioxidant responsive element; CAT = catalase; ERK = extracellular signal-regulated kinase; GCLC = γ-glutamyl-cysteine ligase catalytic subunit; GPx = glutathione peroxidase; GSH = reduced glutathione; GST = glutathione-S-transferase; H_2_O = water molecule; H_2_O_2_ = hydrogen peroxide; HO-1 = heme-oxygenase 1; iNOS = inducible nitric oxide synthase; Keap1 = Kelch-like ECH-associated protein 1; Maf = Musculoaponeurotic fibrosarcoma oncogene homolog; NO• = nitric oxide; O_2_ = oxygen molecule; O_2_•^−^ = superoxide anions; OH• = hydroxyl radicals; ONOO^−^ = peroxynitrite anion; NQO1 = nicotinamide adenine dinucleotide phosphate quinone dehydrogenase 1; Nrf2 = nuclear factor erythroid 2-related factor-2; ROS = reactive oxygen species; RNS = reactive nitrogen species; SOD = superoxide dismutase.

In brief, the evidence shows that quercetin exerts antioxidative properties favouring an increase in osteogenic activities and decrease in osteoclastogenic activities (Figure 4). However, further experiments are recommended to elucidate the exact role of the Nrf2 and ERK signalling pathways in the quercetin-induced upregulation of the antioxidative response in osteoblasts. Meanwhile, investigation into the underlying molecular mechanism governing the antioxidative action of quercetin in osteoclasts is also required.

### 4.5. Anti-Inflammatory Effects

Chronic inflammation is associated with systemic bone loss. The unresolved inflammatory milieu is often characterised by the release of pro-inflammatory factors such as TNF-α, interleukin-1 beta (IL-1β), interleukin-6 (IL-6) and C-reactive protein (CRP), which is not counterbalanced by the production of anti-inflammatory factors such as interleukin-10 (IL-10) and arginase-1 (Arg-1). These inflammatory cytokines represent cell signalling molecules involved in inflammation, and their perturbation implicates abnormal bone metabolism favouring the activation of bone degradation and inhibition of bone formation. The secretion of pro-inflammatory cytokines under inflammatory conditions inhibits osteoblast gene products, partly through the activation of suppressor of mothers against decapentaplegic ubiquitylation regulatory factor 1 (SMURF1), SMURF2 and NF-κB as well as via the suppression of MAPK [126]. Pro-inflammatory mediators also promote Dickkopf-related protein 1 (DKK1) and sclerostin (SOST), inhibiting the Wingless (Wnt)/beta (β)-catenin signalling cascade [126]. Additionally, pro-inflammatory cytokines interact with their respective receptors to stimulate the production of M-CSF and RANKL by osteoblasts, essential for osteoclast proliferation, differentiation and maturation.

In recent years, researchers have investigated whether the bone-sparing actions of quercetin are mediated through its anti-inflammatory properties. An osteoblast and osteoclast co-culture treated with H_2_O_2_ showed an increased level of TNF-α, which was found to be restored to normal level when seeded on hydroxyapatite loaded with quercetin [84]. Another experiment done by the same group of investigators found that quercetin-functionalised hydroxyapatite inhibited IL-6 production on a triculture model consisting of three types of cells, namely human osteoblast-like MG63 cells, osteoclast precursors 2T-110 and human umbilical-vein endothelial cells (HUVECs) [85]. The concentrations of pro- and anti-inflammatory cytokines were also determined in vitro using RAW264.7 cells. Quercetin reduced the levels of IL-1β, TNF-α and IL-6 while increasing those of IL-10 and Arg-1 in RAW264.7 cells exposed to M-CSF, RANKL or LPS [45,46,99]. In vivo, a reduction in TNF-α, IL-6 and CRP levels was observed following quercetin or quercetin-loaded phytosome nanoparticle interventions in ovariectomised [30] and zinc-oxide-nanoparticle-treated rats [44].

**Figure 5:**
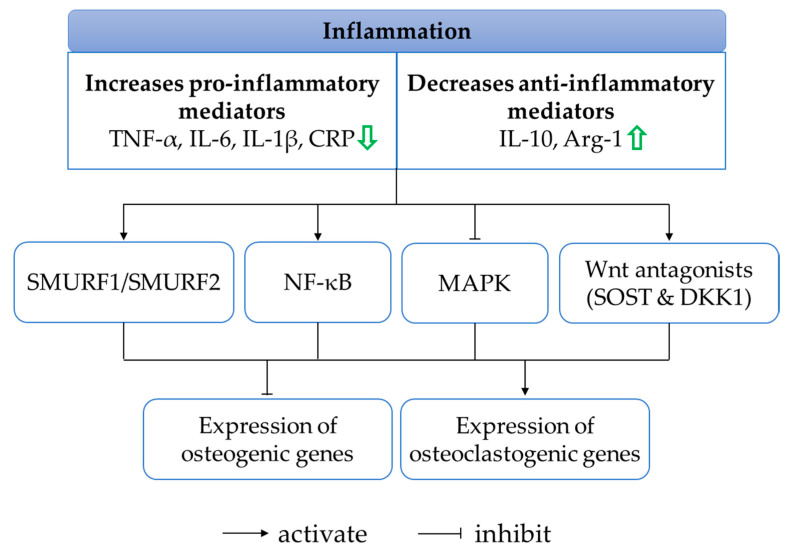
The effects of quercetin on the regulation of inflammatory response in protecting bone. An overwhelming inflammation triggers the activation of SMURF1/SMURF2, NF-κB, inhibition of MAPK and increases production of Wnt antagonists, leading to enhancement of osteogenic genes and suppression of osteoclastogenic genes. Quercetin attenuates the inflammatory response by reducing pro-inflammatory mediators and increasing anti-inflammatory mediators (indicated by the green arrows). Abbreviations: Arg-1 = arginase-1; CRP = C-reactive protein; DKK-1 = Dickkopf-related protein 1; IL-1β = interleukin-1 beta; IL-6 = interleukin-6; IL-10 = interleukin-10; MAPK = mitogen-activated protein kinase; NF-κB = nuclear factor-kappa B; SMURF = suppressor of mothers against decapentaplegic ubiquitylation regulatory factor 1; SOST = sclerostin; TNF-α = tumour necrosis factor-alpha.

Indeed, the process of bone remodelling depends on the tight coupling between pro- and anti-inflammatory mediators. Quercetin helps to resolve overwhelming inflammatory responses by inhibiting pro-inflammatory cytokines and stimulating anti-inflammatory cytokines (Figure 5). The molecular machinery that underlies the anti-inflammatory action of quercetin remains a research gap in the field.

### 4.6. Canonical Wnt/β-Catenin Signalling

The canonical Wnt/β-catenin signalling pathway has a central regulatory role in bone metabolism. In unstimulated conditions, β-catenin is sequestered into a destruction complex consisting of axis inhibition protein 2 (Axin), casein kinase 1 alpha (CK1α), adenomatosis polyposis coli (APC) and glycogen synthase kinase-3 beta (GSK3β). Consequently, β-catenin is phosphorylated, ubiquitinylated and degraded by the proteasome. In the presence of extracellular Wnt protein binding to the Frizzled receptor and low-density-lipoprotein receptor-related protein 5/6 (LRP5/6) co-receptors, the ubiquitination and degradation of β-catenin mediated by GSK3β are inhibited, thus facilitating the accumulation and translocation of β-catenin into the nucleus [127]. The activation of Wnt signalling leads to expression of the Wnt-targeted gene Runx-2, which is essential for osteoblast differentiation [128]. Apart from its well-established role in regulating inflammatory cytokines, the NF-κB signalling pathway has been suggested to interact with the canonical Wnt/β-catenin signalling pathway. A study by Le Henaff et al. (2015) reported that the overactivation of NF-κB transcriptional activity promoted β-catenin phosphorylation and reduced β-catenin target gene expression in mutant osteoblasts. The defective osteoblast differentiation and function were rescued by pharmacological inhibition of NF-κB and activation of Wnt/β-catenin signalling [129].

**Figure 6:**
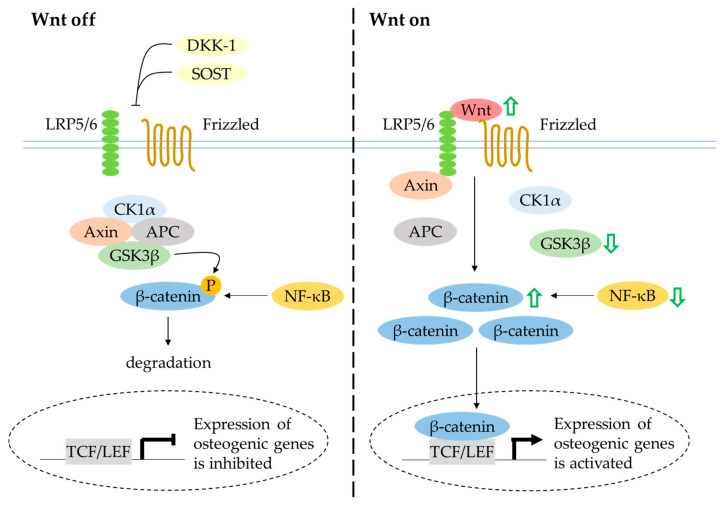
The effects of quercetin on the regulation of Wnt signalling in protecting bone. The activation of Wnt signalling favours the expression of osteogenic genes. Quercetin increases Wnt ligand and inhibits GSK3β, thereby causing accumulation of β-catenin and transcription of Wnt-targeted osteogenic genes (indicated by the green arrows). Abbreviations: APC = adenomatosis polyposis coli; Axin = axis inhibition protein 2; CK1α = casein kinase 1 alpha; DKK-1 = Dickkopf-related protein 1; GSK3β = glycogen synthase kinase-3 beta; LRP5/6 = low-density lipoprotein receptor-related protein 5/6; NF-κB = nuclear factor-kappa B; SOST = sclerostin.

Quercetin rescued LPS-induced impairment of osteogenesis in murine osteoblastic MC3T3-E1 cells by enhancing the protein levels of Wnt3 and β-catenin and decreasing the protein level of GSK3β [75]. Yuan et al. (2018) also found that quercetin protected rat bone-marrow-derived MSCs against TNF-α-induced inhibition of osteoblast differentiation. The activation of NF-κB and degradation of β-catenin were noted in cells treated with TNF-α. These results were reversed after treatment with TNF-α and quercetin [29]. In a preclinical animal experimentation setting, ovariectomy reduced the expression of β-catenin and treatment with isoquercitrin reversed the condition [37]. Taken together, these studies indicate that quercetin potentially orchestrates osteoblast differentiation and function through activation of the canonical Wnt/β-catenin signal transduction pathway (Figure 6). Further investigation into the role of quercetin on Wnt antagonists such as DKK-1 and SOST is required to provide a better understanding of this signalling pathway.

### 4.7. BMP and TGF-β Signalling

BMPs are multifunctional growth factors that belong to the TGF-β superfamily. They bind to a tetrameric receptor complex and transduce intracellular signals via SMAD proteins to express osteoblastogenic genes directly or through Runx-2, suggesting their fundamental roles in skeletal development and bone repair [126]. Another mechanism involved in BMP-2 and TGF-β-mediated osteogenesis is the noncanonical-SMAD-independent pathway (i.e., the MAPK signalling), which phosphorylates Runx-2 and promotes its transcriptional activity [130]. Furthermore, BMP-2 signalling might work dependently with oestrogen-receptor signalling. Oestrogen-stimulated BMP-2 transcription was inhibited by selective oestrogen-receptor-modulator treatment in mouse MSCs [131]. Apart from the well-documented anabolic osteoinductive effects of BMP and TGF-β, they directly support osteoclast formation through the modulation of RANKL expression [132]. Phosphorylated SMAD proteins were detected on bone-marrow-derived osteoclast precursors during RANKL-stimulated differentiation, which was an indicator of BMP activation. The presence of noggin, a BMP antagonist, inhibited RANKL-mediated osteoclast differentiation [133].

The levels of BMP-2 and SMAD proteins and TGF-β1 were evidently increased in rat and mouse bone-marrow-derived MSCs treated with quercetin [34,62,64]. The cross-talk between the BMP-2 and oestrogen-receptor signalling pathways was confirmed by Pang et al. (2018). Their study found that the upregulation of BMP-2 and its downstream targets, as well as other osteogenic genes, by quercetin and oestrogen, was inhibited after the addition of ICI182780 (an oestrogen-receptor antagonist) [64]. In contrast, Yamaguchi and Weitzmann reported that quercetin failed to alleviate the suppressive effects of TNF-α on BMP-2- and TGF-β-induced SMAD activation in MC3T3-E1 cells. However, the osteogenic effects of quercetin were still considered to be due to its anti-NF-κB activity resulting in increased mineralisation in the cultures [74]. Forte and co-researchers reported a slight decrease in TGF-β1 level in the presence of quercetin in an in vitro osteoblast–osteoclast–endothelial cell triculture model. It was noteworthy that the reduction of TGF-β1 level exerted an effect on both osteoblast and osteoclast performance [85].

**Figure 7:**
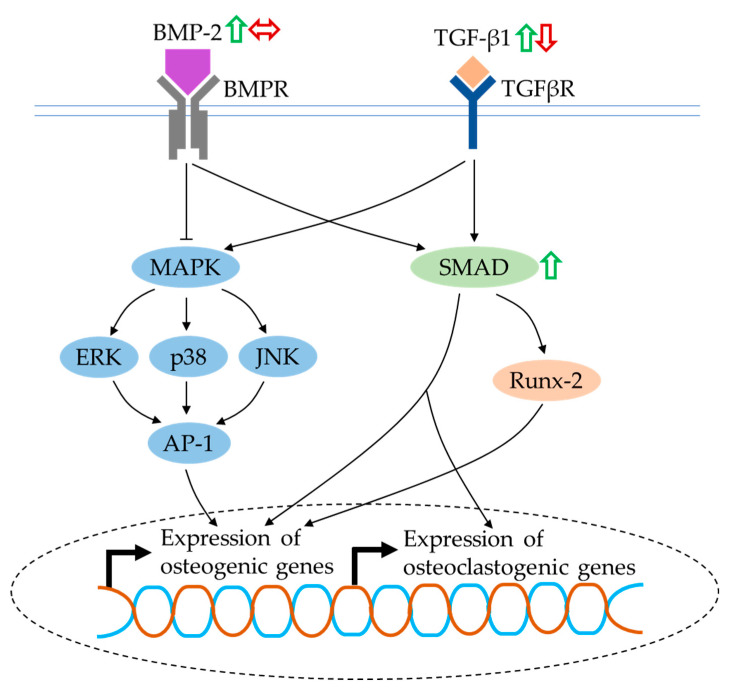
The effects of quercetin on the regulation of BMP and TGF-β signalling in protecting bone. The interaction of BMP-2 and TGF-β with their respective receptors results in the upregulation of osteogenic genes and osteoclastogenic genes via activation of SMAD proteins and/or inhibition of MAPK. Quercetin may cause positive (indicated by the green arrows) or negative effects (indicated by the red arrows) on the levels of BMP-2, TGF-β and SMAD protein in bone cells. Abbreviations: AP-1 = activator protein-1; BMP-2 = bone morphogenetic protein-2; BMPR = bone morphogenetic protein-2 receptor; ERK = extracellular signal-regulated kinase; JNK = c-Jun N-terminal kinase; MAPK = mitogen-activated protein kinase; Runx-2 = runt-related transcription factor 2; SMAD = Suppressor of mothers against decapentaplegic; TGF-β1 = transforming growth factor-beta 1; TGFβR = transforming growth factor-beta receptor

BMP and TGF-β signalling have been demonstrated to positively correlate with osteoblastogenesis and osteoclastogenesis. Quercetin exerts complex competing effects on BMP- and TGF-β-mediated signalling in bone cells (Figure 7). Further studies are recommended to confirm the effects of quercetin on these signalling pathways in osteoclasts.

### 4.8. Regulation of Angiogenesis

Angiogenesis (defined as the formation of new blood vessels) and osteogenesis are the foundational steps of bone regeneration and bone repair. In a bone defect, angiogenesis precedes osteogenesis to provide key elements (such as oxygen, nutrients, minerals, growth factors and bone precursor cells) required for osteogenic processes at the site of injury [134]. Vascular endothelial growth factor (VEGF) is the master player in coupling angiogenesis and osteogenesis, capable of promoting endothelial cells’ migration and proliferation, as well as stimulating the secretion of osteogenic factors [135]. As an angiogenic inducer, angiogenin-1 (ANG-1) also enhances osteoblast differentiation, bone matrix deposition and mineralisation [136]. Basic fibroblast growth factor (bFGF) promotes vascular tissue regeneration, osseous formation and bone remodelling after the implantation of tissue-engineered bone [137]. TGF-β activates VEGF signalling, which is essential for angiogenesis, as well as phosphorylation of SMAD and protein kinase B (Akt), which are required for osteoinduction [138].

**Figure 8:**
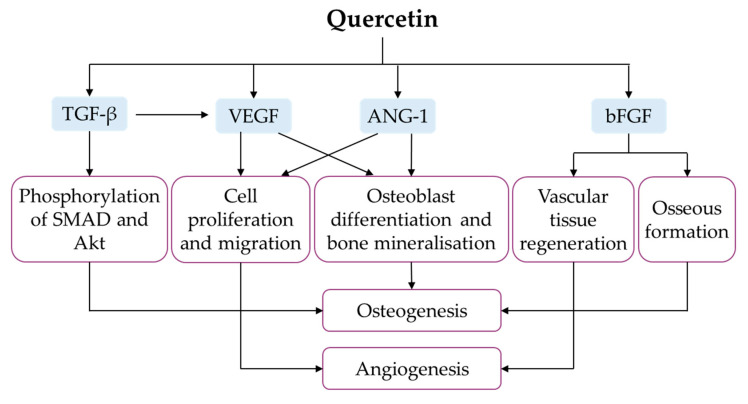
The effects of quercetin on the regulation of angiogenesis. Quercetin increases angiogenic factors (TGF-β, VEGF, ANG-1 and bFGF) to facilitate endothelial-cell proliferation and migration as well as vascular tissue regeneration. Additionally, these angiogenic factors play an important role in promoting osteogenesis. Abbreviations: Akt = protein kinase B; Ang-1 = angiogenin-1; bFGF = basic fibroblast growth factor; SMAD = Suppressor of mothers against decapentaplegic; TGF-β = transforming growth factor-beta; VEGF = vascular endothelial growth factor.

Using MSCs derived from rat bone marrow, Zhou and colleagues conducted two in vitro studies to investigate the effects of quercetin on angiogenic factors. The expressions of VEGF, ANG-1, bFGF and TGF-β were higher in MSCs obtained from bone marrow of normal and ovariectomised rats [34,63]. Mechanistically, Akt was significantly phosphorylated in rat MSCs under quercetin treatment [34]. A quercetin–copper (II) complex was also found to promote angiogenesis, evidenced by increased blood vessel size, length and junctions, in a standard in vivo angiogenesis assay [87]. In animals, quercetin-loaded hydroxyapatite bioceramic microspheres promoted blood-vessel formation after eight weeks in female Sprague–Dawley rats following surgeries for bilateral ovariectomy and femoral bone defect creation [34]. Fayed et al. (2019) reported higher VEGF expression in isoquercitrin-treated ovariectomised rats [37]. Notably, quercetin potentially promotes angiogenesis in vitro and in vivo, leading to the enhancement of bone regeneration (Figure 8).

## 5. Perspectives

The pharmacokinetics of quercetin is an important consideration in understanding its pharmacological effects in vivo and its establishment as an agent for improving bone health. Dietary fat affects the rate and extent of quercetin absorption. A randomised cross-over study found that ingestion of quercetin aglycone together with a standardised formulated high-fat breakfast increased the maximum concentration of total plasma quercetin as compared to those who ate a low-fat breakfast. Hence, the consumption of adequate dietary fat assists the micellisation efficiency of quercetin aglycone in the intestine for absorption [139]. Quercetin has a low oral bioavailability [140]. Generally, ingested flavonoids diffuse passively from the intestinal lumen into enterocytes in unmetabolised form. They can be either metabolised in enterocytes or rapidly transported to the liver for metabolism via phase I and II metabolism. The produced quercetin metabolites are then transported into the systemic circulation for distribution to body tissues [55]. In terms of excretion, the oral clearance of quercetin is rapid, with quertecin having a short half-life in the blood [141]. Various strategies have been employed to overcome these drawbacks and improve the biological performance of quercetin. The use of nanoparticles, microspheres [72], transfersomes [41], hydroxyapatite [68,84,85], transition metals [89] or polyethylene glycolylated graphene oxide [88] functionalised with quercetin helps to promote sustained drug-delivery performance to achieve long-term effects while enhancing rapid osteointegration, bone regeneration and biomechanical properties.

It is noteworthy that higher doses of quercetin exerted either suppressive or lessened activity on the expression of osteoblast-specific genes, osteoblast growth and mineralisation in some of the in vitro studies included [34,47,48,63,64,65,74,76,90,92,93]. The bioactivity of quercetin might depend on the type and position of the sugar attachments of the flavonoid [55]. In other words, a high concentration of quercetin does not always imply its efficacy and effectiveness. Meanwhile, dose-dependent inhibition of osteoclast proliferation and dose-dependent enhancement of osteoclast apoptosis were consistently observed in quercetin-treated cells. In this context, the selection of the optimum dose and type of quercetin to exert bone-protecting effects in humans requires careful consideration and further validation from clinical trials.

Quercetin has been tested in humans for its anti-hypertensive effects [142,143,144,145], lipid-lowering activities [143,145,146] and reduction of upper respiratory tract infections [147]. The dosages used in these clinical trials ranged from 150–1000 mg/day. The safety profile of quercetin has also been evaluated. No notable adverse event was observed in individuals taking up to 5000 mg quercetin daily, characterised by no significant changes in full blood count, metabolic and cholesterol panels, blood coagulation, serum electrolytes, and liver and kidney function [143,148]. In animals, the no-observed-adverse-effect level (NOAEL) for quercetin was found to be 300 mg/kg/day in hamsters and 416 mg/kg/day in rats, which was equivalent to ~2400 mg/day and ~4000 mg/kg for a 60 kg adult human, respectively [149]. Since quercetin has a high NOAEL, it has been presumed that there are no theoretically expected side effects at high doses of quercetin, which requires further validation. However, mild stomach discomfort may develop if quercetin is taken before a meal [148]. Based on its actions on bone, an interventional double-blind placebo randomised controlled study investigated the effects of quercetin supplementation on bone mineralisation biomarkers in patients with type 2 diabetes mellitus. In this study, patients receiving quercetin oral supplement at 500 mg/day for three months had higher levels of serum osteocalcin, calcium and vitamin D compared to their pretreatment values [28]. However, the direct effects of quercetin on bone in osteoporotic patients are still unconfirmed.

## 6. Conclusions

In conclusion, quercetin may represent a useful prophylactic or therapeutic option in the management of skeletal diseases, with the evidence showing that the effects of quercetin on the skeleton are primarily protective. Different types of quercetin formulation have been used, including its free form, derivatives or combined with other compounds. Among these formulations, quercetin derivatives seem to have more potent bone-protective effects than free quercetin and the combination of other phytochemicals may inhibit the anti-osteoporotic effects of quercetin. This review has expanded our understanding of the bone-protecting effects of quercetin, particularly in terms of the regulation of bone mass via control of bone cell differentiation and function. Even though documented works exist to both support and refute the bone-conserving possibility of quercetin, the net effect of quercetin on bone likely depends on the multiple targets influenced by quercetin that modulate the bone microenvironment. Several gold-standard protocol recommendations for future studies are suggested. In in vivo experiments, quercetin at doses lower than the NOAEL can be used to study its beneficial effects on the skeleton. Different routes of administration can be tested for different medical conditions, for instance oral gavage for anti-osteoporotic effects and local injection for anti-fracture effects. Aged animals instead of sexually mature animals might be a more appropriate model. The bone-sparing effects of quercetin must be compared with standard anti-osteoporotic treatments. For cellular studies, three-dimensional co-cultures of osteoblast and osteoclast can be adopted to mimic the in vivo skeletal microenvironment. Secondly, osteoblast cultures should not be subjected to differentiation media for proliferation assays and osteoblast culture should be incubated in differentiation media for differentiation assays. Thirdly, more research in osteocytes is needed, but this is limited to the number of commercial cell lines available. Fourthly, the use of PBMCs from osteoporotic patients is recommended to showcase the effects on osteoclasts more accurately. Human studies investigating the clinical utility of quercetin and its structurally relevant compounds on bone health are limited, thus presenting a subject for further investigation.

## Figures and Tables

**Table 1:** The effects of quercetin on bone in in vivo studies. Symbol ↑ indicates an increase or upregulation, ↓ indicates a decrease or downregulation and ↔ indicates no change.

Type of Animal	Type of Induction	Intervention (Dose, Route and Duration)	Research Findings	References
Female Sprague–Dawley rats	Bilateral ovariectomy	Quercetin (50 mg/kg/day, oral)—8 weeks	Bone mineral density (BMD): ↑; elastic radial degree: ↑; maximum radial degree: ↑; elastic load: ↑; maximum load: ↑; Tb.N: ↑; Tb.Th: ↑	[29]
Female albino rats	Bilateral ovariectomy	Quercetin (50 mg/kg/day, oral)—30 days	ACP: ↓; ALP: ↓; calcium: ↑; phosphorus: ↑; TNF-α: ↓; MDA: ↓; GSH: ↑	[30]
		Quercetin-loaded phytosome nanoparticles (10 or 50 mg/kg, oral)—30 days		
Female C57BL/6J mice	Bilateral ovariectomy	Quercetin (2.5% diet)—4 weeks	Plasma calcium & phosphate: ↔; BMD (total lumbar & total femur): ↑; Cr.Ar: ↑; Cr.Th: ↑; section modulus (rectangular & polar): ↑; BV/TV: ↑; Tb.Th: ↑; Tb.N: ↑; Tb.Sp: ↓; osteoid volume: ↑; osteoid surface: ↑	[31]
Adult female rats	Bilateral ovariectomy	Quercetin (5 mg/kg/day, oral)—12 weeks	BMD (femur epiphysis & proximal tibia): ↑; BV/TV: ↑; Tb.Th: ↔; Tb.Sp: ↓; Tb.N: ↑; femur (Cr.Ar & T.Ar): ↑; tibia (T.Ar, B.Pm & E.Pm): ↑; maximum power: ↔; energy: ↔; stiffness: ↔; osteocalcin: ↓; CTX: ↓	[32]
		Quercetin-6-*C*-β-d-glucopyranoside (5 mg/kg/day, oral)—12 weeks	BMD (femur epiphysis, proximal tibia, L4, femur diaphysis & TFSP): ↑; BV/TV, Tb.Th & Tb.N (femur diaphysis & proximal tibia): ↑; Tb.Sp (femur diaphysis & proximal tibia): ↓; Cr.Ar, Cr.Th, T.Ar, T.Pm, B.Pm & E.Pm (femur & tibia): ↑; maximum power: ↑; energy: ↑; stiffness: ↑; osteocalcin: ↓; CTX: ↓	
Growing female rats	-	Quercetin-6-*C*-β-d-glucopyranoside (5 or 10 mg/kg/day, oral)—12 weeks	MAR: ↑; BFR: ↑; BMD (femur & tibia): ↑; Cr.Ar, Cr.Th, T.Ar, T.Pm, B.Pm & E.Pm (mid-diaphysis & TFSP): ↑	[33]
Adult female rats	Bilateral ovariectomy	Quercetin-6-*C*-β-d-glucopyranoside (5 mg/kg/day, oral)—12 weeks	MAR & BFR (femur diaphysis): ↑; BV/TV, Tb.N, Tb.Th & Conn.D (femur epiphysis): ↑; Tb.Sp, Tb.Pf & SMI (femur epiphysis & proximal tibia): ↓; BV/TV, Tb.N & Conn.D (proximal tibia): ↑	
Female Sprague–Dawley rats	Bilateral ovariectomy + femoral bone defect (size: 3.5 × 4 mm)	Hydroxyapatite bioceramic microspheres loaded with quercetin (200 mM)—8 weeks	BMD: ↑; Tb.Th: ↑; vessel: ↑	[34]
Aged, retired breeder Fischer 344 rats	Bilateral ovariectomy	Quercetin (1000 mg/kg diet) + vitamin D_3_ (2400 IU/kg diet) + genistein (500 mg/kg diet) + resveratrol (200 mg/kg diet)—4 weeks	BMD (whole femur, diaphysis & metaphysis): ↔; BMD (lumbar, L4 & L5): ↔; BV/TV: ↔; BS/BV: ↔; Tb.N: ↔; Tb.Sp: ↔; Tb.Th: ↔; degree of anisotropy: ↔; Conn.D: ↔; osteoclast number: ↔; cortical BV/TV: ↔; Cr.Th: ↔; adipocyte number: ↓	[35]
		Quercetin (2000 mg/kg diet) + vitamin D_3_ (2400 IU/kg diet) + genistein (1000 mg/kg diet) + resveratrol (400 mg/kg diet)—4 weeks		
Female Sprague–Dawley rats	Bilateral ovariectomy	Quercitrin (50, 100 or 200 mg/kg/day, oral)—60 days	BMD (distal femur): ↑; maximum energy absorption, maximum fracture load & stiffness (femoral neck): ↑; calcium: ↑; phosphorus: ↑; ALP: ↑; P1NP: ↑; CTX: ↓; TRAP: ↓; osterix: ↑; Runx-2: ↑	[36]
Female Wistar albino rats	Bilateral ovariectomy	Isoquercitrin (60 mg/kg/day, oral)—8 weeks	Lumbar compression strength: ↑; calcium & phosphorus (serum): ↔; calcium, phosphorus & creatinine (urine): ↓; ALP: ↓; osteocalcin: ↓; VEGF: ↑; β-catenin: ↑; NF-κB: ↓	[37]
Male Wistar albino rats	STZ (50 mg/kg, i.p.)	Quercetin (15 mg/kg/day, i.p.)—4 weeks	Plasma calcium & magnesium: ↑; BV/TV: ↑; Tb.N: ↑; Tb.Th: ↑; load: ↑	[38]
Male Sprague–Dawley rats	STZ (100 mg/kg, i.p.)	Quercetin (30 or 50 mg/kg, oral)—8 weeks	ALP: ↑; osteocalcin: ↑; urinary deoxypyridinoline: ↑; load: ↑; stiffness: ↑; energy absorption: ↑; Young’s modulus: ↑; BMD: ↑; Tb.N: ↑; Tb.Th: ↑; Tb.Sp: ↓; BV/TV: ↑; SMI: ↓; Conn.D: ↑; Cr.Th: ↑; Cr.Ar: ↑; BFR: ↑; MAR: ↑; MS: ↑; 8-OHdG: ↓; total antioxidant capacity: ↑; SOD: ↑; GPx: ↑; CAT: ↑; GST: ↑	[39]
Female Sprague–Dawley rats	Methylprednisolone sodium succinate (40 mg/kg, s.c.)	Quercetin (150 mg/kg, thrice a week, oral)—6 weeks	Bone strength: ↑; osteocalcin: ↑; CTX: ↔; calcium: ↔; phosphorus: ↔; Tb.Th: ↑; Cr.Th: ↑; osteoblast number: ↑; moment of inertia: ↑	[40]
Female Wistar rats	Glucocorticoid methyl prednisolone (40 mg/kg, s.c.)	Quercetin-loaded transfersome film (10 mg/kg/day, topical)—15 days	Calcium: ↑; phosphorus: ↑; ALP: ↓; TRAP: ↓; femur weight: ↑; femur density: ↑; tensile strength: ↑	[41]
Female Sprague–Dawley rats	Bile-duct ligation	Quercetin (150 μmol/kg/day, injection)—4 weeks	osteocalcin: ↑; CTX: ↔; calcium: ↔; phosphorus: ↔; bone strength: ↑; Tb.Th: ↑; Cr.Th: ↑; Cr.Ar: ↑; osteoclast number: ↓; osteoblast number: ↑	[42]
Female fertile Y59 rats	Retinoic acid (80 mg/kg/day, oral)	Quercetin (100 mg/kg/day, oral)—14 days	BMD: ↑; ash, calcium and phosphorus content (femur): ↑; femur weight: ↑; femur length: ↑; MDA: ↓; GSH: ↑	[43]
	-	Quercetin (100 mg/kg/day, oral)—14 days	Ash, calcium and phosphorus content (femur): ↔; femur weight: ↔; femur length: ↔; MDA: ↔; GSH: ↔	
Male Wistar albino rats	Zinc oxide nanoparticles (600 mg/kg/day, 5 days)	Quercetin (200 mg/kg/day, oral)—3 weeks	Bone ALP: ↑; CTX: ↓; calcium: ↔; phosphorus: ↔; magnesium: ↔; NO•: ↓; DNA damage: ↓; TNF-α: ↑; IL-6: ↓; CRP: ↓	[44]
Male BALB/c mice	Titanium-particle-mediated osteolysis	Quercetin (50 or 100 mg/kg, oral)—13 days	Osteolysis: ↓; bone area: ↑; osteoclast number: ↓; PERK: ↓; IRE1: ↓; GRP78: ↓; CHOP: ↓; cleaved caspase-12: ↓; cleaved caspase-3: ↓; Bcl-2: ↑	[45]
Female C57BL/6 mice	Titanium-particle-mediated osteolysis	Quercetin (2 or 5 mg/kg/day)—14 days	BV/TV: ↑; total porosity: ↓; erosion area: ↓; osteoclast number: ↓	[46]
Female Sprague–Dawley rats	Bone defect (diameter: 4 mm)	Quercetin/silk fibroin/hydroxyapatite scaffold with bone-marrow-derived MSCs (implant)—6 weeks	BMD: ↑; BV: ↑; BV/TV: ↑; BS: ↑; Tb.N: ↑; Tb.Th: ↑; bone matrix: ↑; new collagenous tissue: ↑; tissue ingrowth: ↑	[47]
Female Sprague–Dawley rats	Calvarial bone defect (size: 5 × 4 mm)	Quercetin/duck’s foot collagen/hydroxyapatite sponge (25 μM)—8 weeks	BMD: ↑; BV: ↑; new bone formation: ↑	[48]
New Zealand rabbits	Parietal bone defect (size: 5 × 10 mm)	Quercetin solution mixed with collagen matrix—14 days	New bone formation: ↑	[49,50]
Female New Zealand white rabbits	Bone defect	Quercitrin nanocoated implant surface	CTSK: ↓; H^+^ ATPase: ↓; MMP-9: ↓; osteoprotegerin: ↔; RANKL: ↓; IL-10: ↔; TNF-α: ↔; ALP: ↔; LDH: ↔	[51]
Senescence-accelerated OXYS rats	-	Dihydroquercetin (5.06 mg/kg/day) + glucosamine alendronate (1.26 mg/kg/day)—2 months	BMD (total, lumbar and humerus): ↑; F_max_ femur: ↑; femoral strength: ↑; CTX-1: ↓	[52]
Female rabbits	-	Quercetin (10 or 100 μg/kg, 3 times/week, i.m.)—90 days	Cr.Th: ↑	[53]

Abbreviations: 8-OHdG = 8-hydroxy-2′-deoxyguanosine; ACP = acid phosphatase; ALP = alkaline phosphatase; Bcl-2 = B-cell lymphoma 2; BFR = bone formation rate; BMD = bone mineral density; B.Pm = bone perimeter; BS = bone surface; BV = bone volume; CAT = catalase; CHOP = CCAAT/enhancer-binding protein homologous protein; Conn.D = connectivity density; Cr.Ar = cortical bone area; Cr.Th = cortical thickness; CRP = C-reactive protein; CTSK = cathepsin K; CTX = C-terminal telopeptide of type 1 collagen; DNA = deoxyribonucleic acid; E.Pm = endosteal perimeter; GPx = glutathione peroxidase; GRP78 = glucose-regulated protein; GSH = reduced glutathione; GST = glutathione-S-transferase; H^+^ ATPase = vacuolar type proton ATPase; IL-6 = interleukin-6; IL-10 = interleukin-10; IRE1 = inositol-requiring enzyme 1; LDH = lactate dehydrogenase; MAR = mineral apposition rate; MDA = malondialdehyde; MMP-9 = matrix metalloproteinase-9; MS = mineralizing surface; MSCs = mesenchymal stem cells; NF-κB = nuclear factor-kappa B; NO• = nitric oxide; P1NP = N-terminal propeptide of type 1 procollagen; PERK = protein kinase RNA-like endoplasmic reticulum kinase; RANKL = receptor activator of nuclear factor-kappa B ligand; Runx-2 = runt-related transcription factor 2; SMI = structure model index; SOD = superoxide dismutase; T.Ar = periosteal area; Tb.N = trabecular number; Tb.Pf = trabecular pattern factor; Tb.Sp = trabecular separation; Tb.Th = trabecular thickness; TFSP = tibia-fibula separating point; TNF-α = tumour necrosis factor-alpha; T.Pm = periosteal perimeter; TRAP = tartrate-resistant acid phosphatase; TV = total volume; VEGF = vascular endothelial growth factor.

**Table 2:** The effects of quercetin on bone cells. Symbol ↑ indicates an increase or upregulation, ↓ indicates a decrease or downregulation and ↔ indicates no change.

Type of Cell	Intervention	Research Findings	References
Rat bone-marrow-derived MSCs	Quercetin (0.1, 1 or 10 μmol/L)	Cell differentiation: ↑, ALP: ↑, COL1: ↑, osteocalcin: ↑, Cbfα1: ↑, TGF-β1: ↑, BMP-2: ↑, p-ERK1/2: ↑, p-p38: ↑, p-JNK: ↑	[62]
Rat bone-marrow-derived MSCs	Quercetin (1–10 μM)	Cell proliferation: ↑, ALP: ↑, calcium: ↑, Runx-2: ↑, COL1: ↑, BSP: ↑, osteopontin: ↑, osteocalcin: ↑, BMP-2: ↑, VEGF: ↑, ANG-1: ↑, p-ERK: ↑, p-p38: ↑, p-JNK: ↔	[63]
Rat bone-marrow-derived MSCs	Quercetin (1 μM)	ALP: ↑, Runx-2: ↑, COL1: ↑, BSP: ↑, osteopontin: ↑, osteocalcin: ↑, BMP-2: ↑, osteoprotegerin: ↑, RANKL: ↓, VEGF: ↑, ANG-1: ↑, TGF-β: ↑, bFGF: ↑, p-ERK: ↑, p-p38: ↑, p-JNK: ↔, p-Akt: ↑	[34]
Mouse bone-marrow-derived MSCs	Quercetin (0.1–5 μM)	Cell proliferation: ↑, ALP: ↑, mineralised nodules: ↑, Runx-2: ↑, osterix: ↑, osteopontin: ↑, BMP-2: ↑, Smad1: ↑, p-Smad1: ↑, Smad4: ↑, oestrogen receptor signalling: ↑	[64]
Mouse bone-marrow-derived MSCs	Quercetin (25–50 μM)	Cell proliferation: ↑, ALP: ↑, mineralisation: ↑, osteopontin: ↑, Runx-2: ↑, osteocalcin: ↑, osterix: ↑, osteoprotegerin: ↑	[65]
Mouse adipose stem cells	Quercetin (10–100 μM)	Osterix: ↑, Runx-2: ↑, COL1: ↑, BMP-2: ↑, osteopontin: ↑, osteocalcin: ↑	[66]
Human adipose-tissue-derived stromal cells	Quercetin (5 μM)	Osteogenic differentiation: ↑, ALP: ↑, Runx-2: ↑, BMP-2: ↑, osteopontin: ↑, p-ERK: ↑	[67]
Murine osteoblastic MC3T3-E1 cells	Quercetin (10–200 μM)	Cell proliferation: ↑	[68]
	Calcium-deficient hydroxyapatite with quercetin	Osteoblast number: ↑, ALP: ↑, Runx-2: ↑, COL1: ↑, BSP: ↑, osteocalcin: ↑, calcium mineralisation: ↑	
Murine osteoblastic MC3T3-E1 cells	Quercetin (10 μM)	ALP: ↑	[50]
Human osteoblast-like MG-63 cells	Quercetin (1–50 μM)	ALP: ↑, p-ERK: ↑; oestrogen receptor signalling: ↑	[69]
Rat osteoblast-like ROS 17/2.8 cells	Quercetin (5 μM)	BSP: ↑, Cbfα1/Runx-2: ↑	[70]
	Quercetin 3-glucuronide (5 μM)		
Rat femoral-diaphyseal and -metaphyseal tissues	Quercetin (1 or 10 μM)	Calcium content: ↑	[71]
Osteoblasts derived from rat calvaria and bone marrow	Quercetin (10 μM)	BMP-2: ↑, COL1: ↑	[33]
	Quercetin *C*-glucoside (10 or 100 mM)	ALP: ↑, mineralisation: ↑, Runx-2: ↑, BMP-2: ↑, osteocalcin: ↑, COL1: ↑	
Stem-cell spheroids cultured in osteogenic medium	Quercetin (1 μg/mL)	ALP: ↑, Runx-2: ↑	[72]
	Microspheres loaded with quercetin (1 μg/mL)	ALP: ↑, COL1: ↑, Runx-2: ↑	
Human osteoblast-like MG-63 cells	Ethanolic fraction of *Cissus* *quadrangularis* enriched with rutin (65.36 ± 0.75 mg/g) and quercetin (1.06 ± 0.12 mg/g)	ALP: ↑, osteoprotegerin: ↑, RANKL: ↓, RANKL/osteoprotegerin ratio: ↓	[73]
Rat bone-marrow-derived MSCs treated with TNF-α	Quercetin (1 μM)	Cell viability: ↑, calcium nodule formation: ↑, Runx-2: ↑, osterix: ↑, pNF-κB: ↓, β-catenin: ↑	[29]
Murine osteoblastic MC3T3-E1 cells treated with TNF-α	Quercetin (10 μM)	Mineralisation: ↑, NF-κB: ↓, BMP-2- and TGF-β-induced SMAD activation: ↔	[74]
Murine osteoblastic MC3T3-E1 cells treated with LPS	Quercetin (10, 25 or 50 μM)	Mineralisation: ↑, ALP: ↑, osterix: ↑, Runx-2: ↑, osteocalcin: ↑, apoptotic cells: ↓, Bcl-2: ↑, Bcl-XL: ↑, caspase-3: ↓, Bax: ↓, cytochrome c: ↓, Wnt3: ↑, β-catenin: ↑, p-GSK3β: ↓, p-ERK1/2: ↑, p-p38: ↓	[75]
Murine osteoblastic MC3T3-E1 cells treated with LPS	Quercetin (5–10 μM)	ALP: ↑, osterix: ↑, Runx-2: ↑, COL1: ↑, osteocalcin: ↑, BSP: ↑, p-ERK1/2: ↑, p-JNK: ↓	[76]
Murine osteoblastic MC3T3-E1 cells treated with H_2_O_2_ or menadione	Quercetin (1–10 μM)	Cell viability: ↑	[77]
Primary human osteoblasts exposed to cigarette-smoke medium	Quercetin (25, 50 or 100 μM)	Cell viability: ↑, ROS: ↓, HO-1: ↑, SOD: ↑, p-Nrf2: ↑, p-ERK1/2: ↑	[78]
Osteoblasts isolated from fetal rat calvaria	Quercetin aglycone (20 μM)	CAT: ↑, GCLC: ↑, HO-1: ↑, Prdx5: ↑, Nrf2: ↔, p-ERK1/2: ↓, pNF-κB: ↓	[79]
Osteoblasts isolated from fetal rat calvaria treated with H_2_O_2_	Quercetin aglycone (20 μM)	Mineralised nodules: ↑, Runx-2: ↑, ALP: ↑, BSP: ↑, osteocalcin: ↑, GCLC: ↓, HO-1: ↓	[80]
Human osteoblast-like SaOS-2 cells	Quercetin 3-β-D-glucoside (0.1 or 0.3 μM) + polyphosphate (3–100 μM)	Runx-2: ↑, ATF6: ↑, Ets1: ↑	[81]
Murine osteoblastic MC3T3-E1 cells treated with H_2_O_2_	Quercitrin (1 μg/mL)	Cell growth: ↑, collagen: ↑, ALP: ↑, mineralisation: ↑, RANKL: ↓, MDA: ↓, protein carbonyl: ↓, nitrotyrosine: ↓	[82]
Murine osteoblastic MC3T3-E1 cells	Quercitrin (200 or 500 μM)	BSP: ↑, osteocalcin: ↑, osteoprotegerin: ↔, RANKL: ↓	[83]
Bone-marrow-derived MSCs	Quercetin/silk fibroin/hydroxyapatite scaffold	Cell growth and proliferation: ↑, ALP: ↑, COL1: ↑, osteocalcin: ↑, Runx-2: ↑	[47]
Bone-marrow-derived MSCs	Quercetin/duck’s foot collagen/hydroxyapatite sponge (25 μM)	Cell proliferation: ↑, ALP: ↑, COL1: ↑, osteocalcin: ↑, Runx-2: ↑	[48]
Co-culture model containing human osteoblast-like MG63 cells and osteoclast precursors 2T-110 treated with H_2_O_2_	Quercetin-loaded hydroxyapatite	Cell viability of osteoblast: ↑, osteocalcin: ↑, Runx-2: ↑, LDH: ↓, TNF-α: ↓, ROS: ↓, cell viability of osteoclast: ↓, caspase 3: ↑	[84]
Triculture model containing human osteoblast-like MG63 cells, osteoclast precursors 2T-110 and HUVECs	Quercetin-loaded hydroxyapatite	Cell proliferation of osteoblast: ↑, ALP: ↑, COL1: ↑, osteocalcin: ↑, osteonectin: ↑, cell proliferation of osteoclast: ↓, osteoprotegerin: ↑, RANKL: ↓, osteoprotegerin/RANKL ratio: ↑, CTSK: ↓, TGF-β1: ↓, IL-6: ↓	[85]
Murine osteoblastic MC3T3-E1 cells	Quercetin vanadyl (IV) complexes	ALP: ↑, COL1: ↑, p-ERK: ↑	[86]
Human osteoblast-like MG-63 cells	Quercetin–copper (II) complexes (20–60 μM)	ALP: ↑, mineralised matrix: ↑, calcium deposition: ↑, Runx-2: ↑, COL1: ↑, pre-mir-15b: ↑, blood vessel size, length and junctions: ↑	[87]
MSCs	ADM-GO-PEG/quercetin (10 μM) scaffold	Cell proliferation: ↑, lipoprotein lipase: ↑, peroxisome proliferator-activated receptor-γ: ↑, ALP: ↑, Runx-2: ↑	[88]
Bone-marrow-derived MSCs	Quercitrin-nanocoated titanium surfaces	Cell viability: ↑, cell adhesion: ↑, calcium content: ↑, mineralisation: ↑	[89]
Murine osteoblastic MC3T3-E1 cells treated with TNF-α	Quercetin (1–10 μM)	Cell viability: ↓, cytotoxicity: ↑, apoptosis: ↑, Fas activation: ↑, PARP cleavage: ↑, Bcl-2: ↓, cytochrome c: ↓, degradation of procaspase-8: ↑, caspase-8: ↑, caspase-3: ↑, AP-1 activity: ↑, p-JNK: ↑	[90,91]
Rat calvarial osteoblast-like cells	Quercetin (0.1–10 μM)	Cell proliferation: ↓, ALP: ↓, osteocalcin: ↓, deposition of calcium: ↓, mineralised nodules: ↓,	[92]
MSCs induced to differentiate into osteoblasts	Quercetin (10 μM)	Cell proliferation: ↓, ALP: ↓, mineralisation: ↓, COL1: ↓, osteocalcin: ↓	[93]
RAW264.7 cells treated with M-CSF and RANKL	Quercetin (6.3 or 25 μmol/L)	Area of osteoclast: ↓, TRAP-positive cells: ↓, bone resorption area: ↓, F-actin ring area and number: ↓, c-Fos: ↓, NFATc1: ↓, MMP-9: ↓, CTSK: ↓, IL-1β: ↓, TNF-α: ↓, IL-6: ↓, IL-10: ↑, Arg-1: ↑, iNOS: ↓	[46]
RAW264.7 cells treated with RANKL	Quercetin (10 μM)	TRAP-positive multinucleated cells: ↓, c-Fos: ↓, RANK: ↓, CalcR: ↓	[94]
RAW264.7 cells treated with M-CSF and RANKL	Quercetin (2–5 μM)	Osteoclast formation: ↓, pit formation: ↓, disruption of actin ring: ↑, TRAP activity: ↓	[95]
RAW264.7 cells treated with RANKL	Quercetin (0.1–25 μM)	Osteoclast number: ↓, NF-κB: ↓	[74]
RAW264.7 cells treated with RANKL	Quercetin (40–160 μmol/L)	Osteoclast number: ↓, cell apoptosis: ↓, PERK: ↓, IRE1: ↓, GRP78: ↓, CHOP: ↓, caspase-12: ↓, caspase-3: ↓, Bcl-2: ↑, TNF-α: ↓, IL-1β: ↓, IL-6: ↓, TRAP: ↓, RANK: ↓	[45]
RAW264.7 cells treated with RANKL	Quercetin (1–10 μM)	TRAP-positive multinucleated cells: ↓, CalcR: ↓, CTSK: ↓, MMP-9: ↓, NFATc1: ↓	[31]
	Quercetin-3-*O*-β-D-glucuronide (1–10 μM)	TRAP-positive multinucleated cells: ↓	
RAW264.7 cells treated with RANKL	Quercetin (10–200 μM)	Cell proliferation: ↓, osteoclast number: ↓	[68]
	Calcium-deficient hydroxyapatite with quercetin	Cell proliferation: ↓, osteoclast number: ↓, TRAP activity: ↓	
RAW264.7 cells treated with RANKL	Quercetin (1–10 μM)	TRAP-positive multinucleated cells: ↓, TRAP: ↓, NF-κB activation: ↓, AP-1 activation: ↓	[96]
Human PBMCs treated with M-CSF and RANKL		Osteoclast number: ↓, resorbed area: ↓, hydroxylysylpyridinoline: ↓	
Bone-marrow macrophages treated with M-CSF and RANKL	Quercetin (10 or 20 μM)	TRAP-positive multinucleated cells: ↓, RANK: ↓, c-Fos: ↓	[32]
	Quercetin-6-*C*-β-d-glucopyranoside (1 or 100 nM)	TRAP-positive multinucleated cells: ↓, RANK: ↓, c-Fos: ↓	
Highly purified rabbit osteoclasts	Quercetin (50 μM)	Resorption pit area: ↓, hydroxylysylpyridinoline: ↓, apoptotic osteoclast: ↑, ROS: ↓	[97]
RAW264.7 cells treated with LPS	Quercetin (15, 25 or 50 μM)	Osteoclast number: ↓, TRAP: ↓, MMP-9: ↓, CTSK: ↓, RANK: ↓, COX-2: ↓, TRAF6: ↓, p38 MAPK: ↑, p-JNK: ↑, number of apoptotic cells: ↑, Bax: ↑, Bcl-2: ↓, number of pits: ↓	[98]
Mouse bone-marrow cells treated with PTH	Quercetin (0.01–1 μM)	Osteoclast number: ↓	[71]
RAW264.7 cells treated with LPS	Quercetin (0.03–3 μg/mL)	NO: ↓, ROS: ↓, TNF-α: ↓, IL-1β: ↓, IL-6: ↓	[99]
	Quercitrin (0.045–4.5 (0.03–3 μg/mL)		
RAW264.7 cells treated with RANKL	Quercitrin (200 or 500 μM)	TRAP-positive multinucleated cells: ↓, resorption pit: ↓, TRAP: ↓, CTSK: ↓, alpha v intergrin: ↑, MMP-9: ↓, H^+^ ATPase: ↓, Dc-Stamp: ↓	[83]
RAW264.7 cells treated with RANKL	Quercitrin-nanocoated implant surface	TRAP: ↓, CalcR: ↓, CTSK: ↓, H^+^ ATPase: ↓, MMP-9: ↓	[51]

Abbreviations: Akt = protein kinase B; ALP = alkaline phosphatase; ANG-1 = angiogenin-1; AP-1 = activator protein-1; Arg-1 = arginase-1; ATF6 = activating transcription factor 6; Bax = Bcl-2-associated X protein; Bcl-2 = B-cell lymphoma 2; Bcl-XL = B-cell lymphoma-extra large; bFGF = basic fibroblast growth factor; BMP-2 = bone morphogenetic protein-2; BSP = bone sialoprotein; CalcR = calcitonin receptor; CAT = catalase; Cbfα1 = core binding factor alpha 1; c-Fos = cellular proto-oncogene; C−P = CCAAT/enhancer-binding protein homologous protein; COL1 = type 1 collagen; CTSK = cathepsin K; Dc-Stamp = dendritic cell-specific transmembrane protein; ERK = extracellular signal-regulated kinase; Ets1 = Ets oncogene homolog 1; GCLC = γ-glutamyl-cysteine ligase catalytic subunit; GRP78 = glucose-regulated protein; GSK3β = glycogen synthase kinase-3 beta; H+ ATPase = vacuolar type proton ATPase; HO-1 = heme-oxygenase 1; IL-1β = interleukin-1 beta; IL-6 = interleukin-6; IL-10 = interleukin-10; iNOS = inducible nitric oxide synthase; IRE1 = inositol-requiring enzyme 1; JNK = c-Jun N-terminal kinase; LDH = lactate dehydrogenase; MAPK = mitogen-activated protein kinase; MDA = malondialdehyde; MMP-9 = matrix metalloproteinase-9; MSCs = mesenchymal stem cells; NFATc1 = nuclear factor of activated T-cells cytoplasmic 1; NF-κB = nuclear factor-kappa B; NO• = nitric oxide; Nrf2 = nuclear factor erythroid 2-related factor-2; Ob.N = osteoblast number; PARP = poly(ADP-ribose) polymerase; PERK = protein kinase RNA-like endoplasmic reticulum kinase; Prdx5 = peroxiredoxin-5; PTH = parathyroid hormone; RANK = receptor activator of nuclear factor-kappa B; RANKL = receptor activator of nuclear factor-kappa B ligand; ROS = reactive oxygen species; Runx-2 = runt-related transcription factor 2; SMAD = suppressor of mothers against decapentaplegic; SOD = superoxide dismutase; TGF-β = transforming growth factor-beta; TNF-α = tumour necrosis factor-alpha; TRAF6 = tumour necrosis factor receptor-associated factor 6; TRAP = tartrate-resistant acid phosphatase; VEGF = vascular endothelial growth factor; Wnt = Wingless.

## Abbreviations

8-OHdG	8-hydroxy-2′-deoxyguanosine
ACP	Acid phosphatase
Akt	Protein kinase B
ALP	Alkaline phosphatase
ANG-1	Angiogenin-1
AP-1	Activator protein-1
Apaf-1	Apoptotic protease activating factor-1
APC	Adenomatosis polyposis coli
ARE	Antioxidant responsive element
Arg-1	Arginase-1
ATF6	Activating transcription factor 6
Axin	Axis inhibition protein 2
Bak	Bcl-2 homologous antagonist/killer
Bax	Bcl-2-associated X protein
Bcl-2	B-cell lymphoma 2
Bcl-XL	B-cell lymphoma-extra large
bFGF	Basic fibroblast growth factor
BFR	Bone formation rate
BMD	Bone mineral density
BMP-2	Bone morphogenetic protein-2
B.Pm	Bone perimeter
BS	Bone surface
BSP	Bone sialoprotein
BV	Bone volume
CalcR	Calcitonin receptor
CAT	Catalase
Cbα1	Core binding factor alpha 1
c-Fos	Cellular proto-oncogene
CHOP	CCAAT/enhancer-binding protein homologous protein
CK1α	Casein kinase 1 alpha
COL1	Type 1 collagen
Conn.D	Connectivity density
Cr.Ar	Cortical bone area
Cr.Th	Cortical thickness
CRP	C-reactive protein
CTSK	Cathepsin K
CTX	C-terminal telopeptide of type 1 collagen
dATP	Deoxyadenosine triphosphate
Dc-Stamp	Dendritic cell-specific transmembrane protein
DISC	Death-inducing signalling complex
DKK1	Dickkopf-related protein 1
DNA	Deoxyribonucleic acid
E.Pm	Endosteal perimeter
ERK	Extracellular signal-regulated kinase
Ets1	Ets oncogene homolog 1
FADD	Fas-associated death domain
FasL	Fas ligand
GCLC	γ-glutamyl-cysteine ligase catalytic subunit
GPx	Glutathione peroxidase
GRP78	Glucose-regulated protein
GSH	Reduced glutathione
GSK3β	Glycogen synthase kinase-3 beta
GST	Glutathione-S-transferase
H_2_O	Water molecule
H_2_O_2_	Hydrogen peroxide
H^+^ ATPase	Vacuolar type proton ATPase
HO-1	Heme-oxygenase 1
HUVECs	Human umbilical vein endothelial cells
IL-1β	Interleukin-1 beta
IL-6	Interleukin-6
IL-10	Interleukin-10
i.m.	Intramuscular
iNOS	Inducible nitric oxide synthase
i.p.	Intraperitoneal
IRE1	Inositol-requiring enzyme 1
JNK	c-Jun N-terminal kinase
Keap1	Kelch-like ECH-associated protein 1
LDH	Lactate dehydrogenase
LPS	Lipopolysaccharides
LRP5/6	Low-density lipoprotein receptor-related protein 5/6
Maf	Musculoaponeurotic fibrosarcoma oncogene homolog
MAP2K	Mitogen-activated protein kinase kinase
MAP3K	Mitogen-activated protein kinase kinase kinase
MAPK	Mitogen-activated protein kinase
MAR	Mineral apposition rate
M-CSF	Macrophage colony-stimulating factor
MDA	Malondialdehyde
MMP-9	Matrix metalloproteinase-9
MS	Mineralising surface
MSCs	Mesenchymal stem cells
N_2_O_3_	Dinitrogen trioxide
NFATc1	Nuclear factor of activated T-cells cytoplasmic 1
NF-κB	Nuclear factor-kappa B
NO•	Nitric oxide
NO_2_•	Nitrogen dioxide
NQO1	Nicotinamide adenine dinucleotide phosphate quinone dehydrogenase 1
Nrf2	Nuclear factor erythroid 2-related factor-2
O_2_	Oxygen molecule
O_2_•^−^	Superoxide anions
OH•	Hydroxyl radicals
ONOO^-^	Peroxynitrite anion
P1NP	N-terminal propeptide of type 1 procollagen
PARP	Poly(ADP-ribose) polymerase
PBMCs	Peripheral-blood monocytic cells
PERK	Protein kinase RNA-like endoplasmic reticulum kinase
Prdx5	Peroxiredoxin-5
PTH	Parathyroid hormone
RANK	Receptor activator of nuclear factor-kappa B
RANKL	Receptor activator of nuclear factor-kappa B ligand
RNS	Reactive nitrogen species
ROS	Reactive oxygen species
Runx-2	Runt-related transcription factor 2
s.c.	Subcutaneous
SMAD	Suppressor of mothers against decapentaplegic
SMI	Structure model index
SMURF1	Suppressor of mothers against decapentaplegic ubiquitylation regulatory factor 1
SOD	Superoxide dismutase
SOST	Sclerostin
STZ	Streptozotocin
T.Ar	Periosteal area
Tb.N	Trabecular number
Tb.Pf	Trabecular pattern factor
Tb.Sp	Trabecular separation
Tb.Th	Trabecular thickness
TFSP	Tibia–fibula separating point
TGF-β	Transforming growth factor-beta
TNF-α	Tumour necrosis factor-alpha
T.Pm	Periosteal perimeter
TRAF6	Tumour necrosis factor receptor-associated factor 6
TRAIL	Tumour necrosis factor-related apoptosis-inducing ligand
TRAP	Tartrate-resistant acid phosphatase
TV	Total volume
VEGF	Vascular endothelial growth factor
Wnt	Wingless
